# Synthesis, Biological Profiling and Determination of the Tubulin-Bound Conformation of 12-Aza-Epothilones (Azathilones)

**DOI:** 10.3390/molecules21081010

**Published:** 2016-08-03

**Authors:** Andrea Jantsch, Lidia Nieto, Jürg Gertsch, Javier Rodríguez-Salarichs, Ruth Matesanz, Jesús Jiménez-Barbero, J. Fernando Díaz, Ángeles Canales, Karl-Heinz Altmann

**Affiliations:** 1 Department of Chemistry and Applied Biosciences, Insitute of Pharmaceutical Sciences, ETH Zürich, CH-8093 Zürich, Switzerland; a.jantsch@hotmail.com; 2 Department of Organic Chemistry I, Fac. C. C. Químicas, Universidad Complutense de Madrid, ES-28040 Madrid, Spain; linietog@gmail.com (L.N.); angelescm@cib.csic.es (Á.C.); 3 Centro de Investigaciones Biológicas, Consejo Superior de Investigaciones Científicas, 28040 Madrid, Spain; javierr@cib.csic.es (J.R.-S.); ruth.m.r@cib.csic.es (R.M.); fer@cib.csic.es (J.F.D.); 4 Institute of Biochemistry and Molecular Medicine, University of Bern, CH-3012 Bern, Switzerland; juerg.gertsch@ibmm.unibe.ch; 5 CIC bioGUNE, 48170 Derio, Spain; jjbarbero@cicbiogune.es; 6 Ikerbasque, Basque Foundation for Science, 48009 Bilbao, Spain; 7 Department of Organic Chemistry II, Faculty of Science & Technology, University of the Basque Country, 48940 Leioa, Bizkaia, Spain

**Keywords:** anticancer, azathilones, conformation, drug discovery, epothilones, natural product, SAR, STD, synthesis, tubulin

## Abstract

12-Aza-epothilones (azathilones) incorporating quinoline side chains and bearing different N12-substituents have been synthesized via highly efficient RCM-based macrocyclizations. Quinoline-based azathilones with the side chain N-atom in the *meta*-position to the C15 atom in the macrocycle are highly potent inhibitors of cancer cell growth in vitro. In contrast, shifting the quinoline nitrogen to the position *para* to C15 leads to a ca. 1000-fold loss in potency. Likewise, the desaturation of the C9-C10 bond in the macrocycle to an *E* double bond produces a substantial reduction in antiproliferative activity. This is in stark contrast to the effect exerted by the same modification in the natural epothilone macrocycle. The conformation of a representative azathilone bound to α/β-tubulin heterodimers was determined based on TR-NOE measurements and a model for the posture of the compound in its binding site on β-tubulin was deduced through a combination of STD measurements and CORCEMA-ST calculations. The tubulin-bound, bioactive conformation of azathilones was found to be overall similar to that of epothilones A and B.

## 1. Introduction

**Figure 1:**
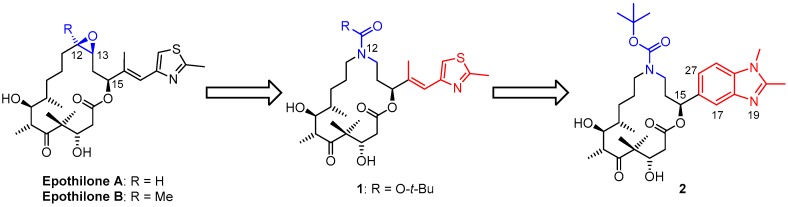
Molecular structures of epothilones A and B and their evolution into azathilones.

Epothilones A and B (Figure 1) are the major representatives of a larger family of natural products that were first isolated from the myxobacterium *Sorangium cellulosum* by Reichenbach and Höfle in 1987 [1,2]. Epothilones A and B are highly active microtubule-stabilizing agents [3,4] and they both show potent in vitro antiproliferative activity [3,4,5], against both drug-sensitive as well as multidrug-resistant cancer cell lines; in addition, for epothilone B excellent in vivo antitumor activity has been demonstrated in tumor xenograft models in mice [5,6,7]. Based on these preclinical findings, the epothilone scaffold has been widely explored in anticancer drug discovery [8,9] and at least nine epothilone analogs or derivatives have entered clinical trials in humans. This includes the epothilone B lactam ixabepilone, which was approved by the US FDA in 2007 for the treatment of advanced and drug-resistant breast cancer [10]. Quite intriguingly, however, the structural diversity within this substantial group of clinical candidates is rather limited, which could restrict the potential for pharmacological differentiation between these compounds. In order to address this issue, we have extensively investigated a number of what we have termed hypermodified epothilone analogs, i.e., analogs that are of only limited structural similarity with the original natural products [11]. While the early part of these studies had targeted analogs that were still based on a regular polyketide backbone throughout [12,13,14], our more recent work has focused on structures where carbon 12 has been replaced by an acylated nitrogen atom, thus leading to 12-aza-epothilones or azathilones [15,16,17] (Figure 1). In these structures, the regular polyketide pattern that originates from the successive assembly of (substituted) C2 units in the course of the biosynthesis of epothilones is disrupted by the incorporation of nitrogen in place of the α-carbon of the propionate or acetate unit from which C26 (in the case of epothilone B), C12 and C13 are derived (for numbering see Figure 1). Thus, while most of the gross structural features of azathilones undoubtedly resemble those of polyketide-derived macrolides, they may in fact be designated as “non-natural” natural products [18], as they could not be the product of a canonical biosynthesis pathway.

The initial design of the azathilones was exclusively chemistry-driven, the basic objective being the discovery of analogs that would be synthetically more readily accessible than the natural products themselves. At the same time, and somewhat simplistically, the carbonyl oxygen of the N12 acyl residue of azathilones was meant to mimic the epoxide oxygen in natural epothilones in their interactions with their purported target protein tubulin (we use the term “purported target”, as it was not clear at this point if the compounds would in fact display the same mode of action as natural epothilones). This (weak) structural hypothesis became obsolete shortly after the beginning of our synthetic work on azathilones, when it was shown that epothilones C and D, which incorporate a 12,13-*Z* double bond instead of an epoxide moiety, were equally potent microtubule-stabilizing agents as the corresponding parent compounds epothilones A and B, respectively [19,20]. Likewise, it was found that the epoxide moiety in epothilones A and B could be replaced by a cyclopropane ring without any loss in microtubule-stabilizing activity or cellular potency [21,22]. Nevertheless, and independent of the exact role of the carbonyl oxygen of the N12 acyl substituent, our first series of azathilones with R = Me, Et, and *t*Bu (Figure 1), while clearly less active than natural epothilones, showed significant antiproliferative activity against the human cervical carcinoma cell line KB31 (IC_50_ values between 70 and 200 nM) [15]. However, the most potent compound that emerged from our initial work was the N12-*tert*-butyl carbamate **1** (Figure 1, R = O-*t*Bu), which was an intermediate in the synthesis of the above N12-acyl analogs and which was found to be only ca. 15-fold less active against KB31 cells than epothilone A (IC_50_ = 31 nM vs. 2.1 nM for epothilone A) [15]. In addition, analog **1**, like other urethane-based azathilones, appeared to be less susceptible to Pgp-mediated drug efflux than the corresponding amides. Building on these early findings we then explored if the potency of **1** could be improved by the replacement of the natural thiazolyl-vinyl side chain by a dimethylbenzimidazole moiety (Figure 1, analog **2**) [16], a modification which we had previously shown to lead to enhanced antiproliferative activity for polyketide-based epothilone analogs [23,24,25]. While we had hoped that this would also be the case for **2** in comparison with **1**, we were in fact stunned by the magnitude of the effect observed. Thus, the IC_50_ value of **2** against KB31 cells was ca. 90-fold lower than for **1** (0.34 nM vs. 31 nM) and similar differences were also observed for the A549 lung carcinoma, MCF-7 breast carcinoma, and PC3 prostate carcinoma cell lines [16]. These differences are significantly higher than those observed between (otherwise identical) polyketide-based epothilone analogs bearing either a thiazolyl-vinyl or a dimethylbenzimidazole side chain [23,24,25]. Equally intriguing is the fact that the desaturation of the C9-C10 bond in **1** or **2** to a *trans* double bond is associated with a profound loss in potency [16]; this is fundamentally different from the effect observed for the same modification in epothilone analogs that are based on a regular polyketide-derived macrolactone ring [14,26,27].

**Figure 2:**
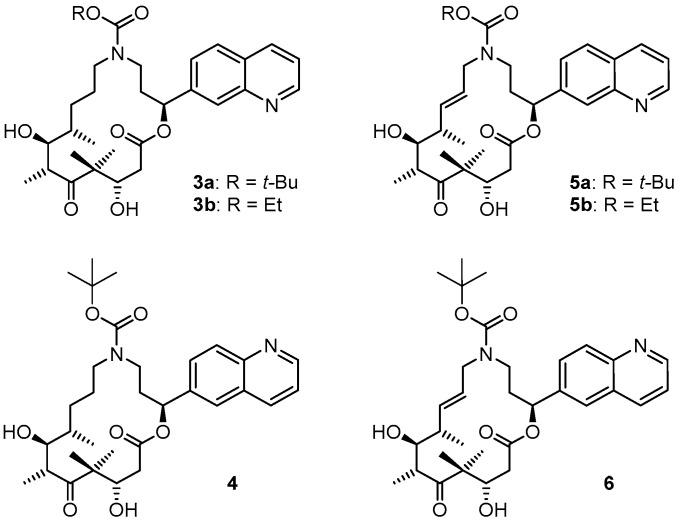
Molecular structures of azathilones investigated in this study.

Azathilone **2** promotes tubulin assembly in vitro with similar potency as epothilone A and its effects at the cellular level are typical of a microtubule-stabilizing agent [16], which clearly shows that the transition from the regular epothilone scaffold to an azathilone-type macrocycle does not lead to a switch in the mode of action. However, a question that has remained unaddressed so far is, if the profound antiproliferative activity of **2** is uniquely linked to its particular dimethylbenzimidazole side chain or if related analogs with other benzo-fused heterocyclic side chain moieties would retain similar activity, as is the case for the corresponding epothilone B and D analogs [25]. Likewise, it has not been determined if such alternative side chain modifications would be associated with the same loss in potency as **1** and **2** upon desaturation of the C9-C10 bond to a *E* double bond and if these effects would depend on the position of the *N*-atom in the heterocycle. In order to address these questions, we have investigated a series of quinoline-based azathilones **3**–**6** (Figure 2) and we have determined their affinity to cross-linked microtubules and their in vitro antiproliferative activity against three different human cancer cell lines.

In addition, we have also performed extensive NMR studies with **2**, in order to characterize its interactions with the tubulin/microtubule system at the molecular level and thus shed light on the distinct difference in activity between **2** and its 9,10-*E*-dehydro congener.

## 2. Results and Discussion

### 2.1. Chemistry

**Scheme 1:**
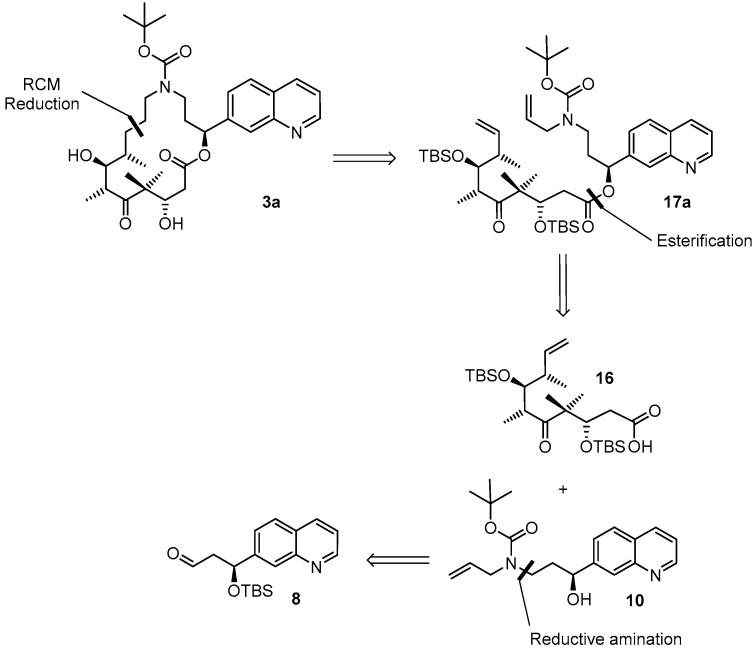
Retrosynthesis of azathilone **3a**.

As part of our previous work, we had developed two strategies for the synthesis of azathilone **2** that were based on macrocyclization either by macrolactonization or by ring-closing olefin metathesis (RCM) between C9 and C10 [16]. The former approach was elaborated for scale-up purposes in response to difficulties that had been encountered for the reduction of the 9,10 double bond formed in the ring-closing step. In spite of these difficulties, our synthesis of quinoline-based azathilones **3** and **4** was to rely on RCM-based macrocyclization, simply because this approach would also provide simultaneous access to the desired 9,10-dehydro analogs **5** and **6** [28]. We also felt that a sufficient investment in the optimization of the reduction step after RCM would eventually allow us to perform the reaction in satisfactory yield. The corresponding general retrosynthesis of **3**–**6** is exemplified in Scheme 1 for target structure **3a**. Thus, **3a** would be obtained from diene **17a** by RCM followed by deprotection and reduction. Diene **17a** in turn would be assembled by esterification of known acid **16** [29] with alcohol **10**. The latter would be derived from aldehyde **8** [25] by reductive amination with allyl amine followed by carbamoylation with BOC-anhydride.

**Scheme 2:**
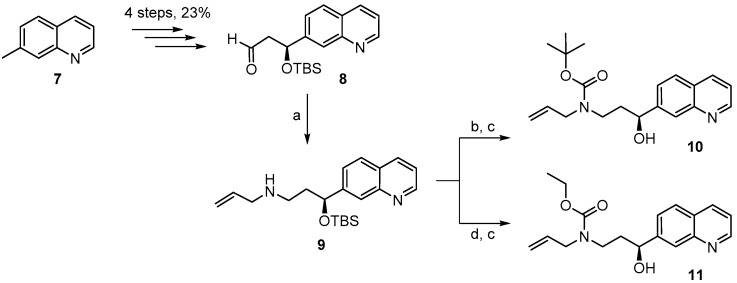
Synthesis of alcohols **10** and **11**. *Reagents and Conditions*: (**a**) i. AllylNH_2_, MS 4 Å, THF, rt → 50 °C, 24 h; ii. NaBH_4_, MeOH, 0 °C, 20 min, 76% (2 steps); (**b**) Boc_2_O, CH_2_Cl_2_, rt, 14 h, 86%; (**c**) TBAF, THF, rt, 5–7 h; **10**: quant; **11**: 96%; (**d**) EtOC(O)Cl, acetone, rt, 2 h, 87%.

In the forward direction, the reductive amination of **8** with allyl amine proceeded smoothly to provide amine **9** in 76% yield (Scheme 2). Subsequent reaction of **9** with BOC-anhydride or ethyl chloroformate then gave the respective carbamates, which were further transformed into the free alcohols **10** and **11** by TBS-ether cleavage with TBAF in THF in 86% and 84% overall yield, respectively.

**Scheme 3:**
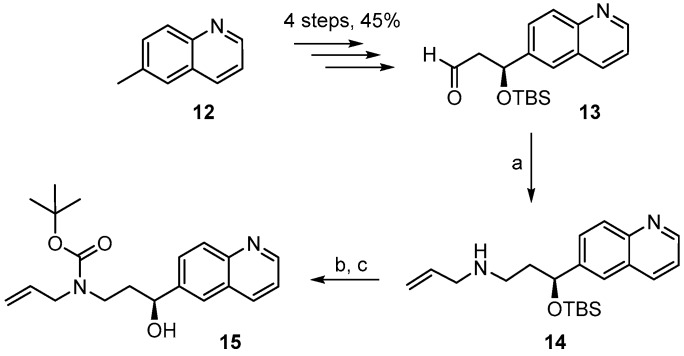
Synthesis of alcohol **15**. *Reagents and Conditions*: (**a**) i. AllylNH_2_, MS 4 Å, THF, rt to 50 °C, 24 h; ii. NaBH_4_, MeOH, 0 °C, 20 min, 67% (2 steps); (**b**) Boc_2_O, CH_2_Cl_2_, rt, 14 h, 86%; (**c**) TBAF, THF, rt, 5 h, 95%.

As illustrated in Scheme 3, alcohol **15** was obtained from aldehyde **13** [30] in analogy to the synthesis of **10** from **8** in 55% overall yield.

**Scheme 4:**
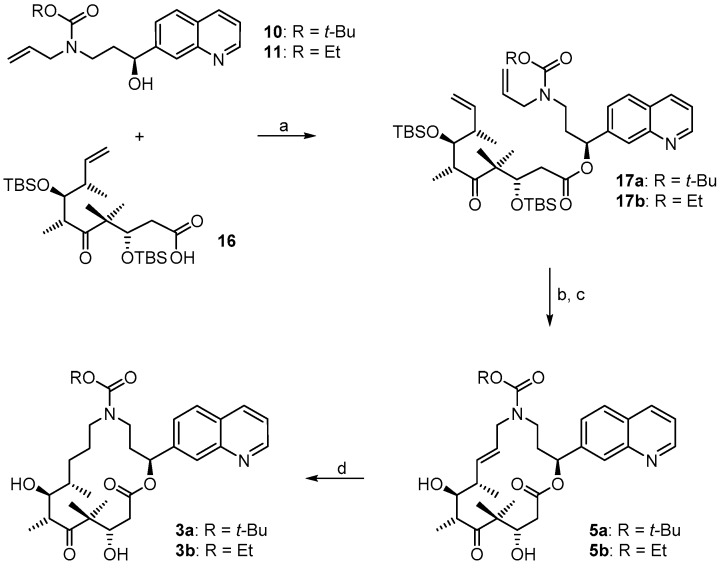
Synthesis of azathilones **5a/b** and **3a/b**. *Reagents and Conditions*: (**a**) EDC, DMAP, CH_2_Cl_2_, 0 °C → rt, 5 h, **17a**: 84%; **17b**: 84%; (**b**) Grubbs II (15 mol %), toluene, refl, 30 min, 82% (R = *t*Bu); 96% (R = Et); (**c**) HF∙py, pyridine, THF, 0 °C → rt, 8 h, **5a**: 67%; **5b**: 51% (after HPLC purification); (**d**) PADA, CH_2_Cl_2_, AcOH, rt, 5.5–7 days. **3a**: 66%; **3b**: 65% (after HPLC purification).

The EDC/DMAP-mediated esterification of acid **16** with alcohols **9** and **10** furnished dienes **17a/b** in high yields (84% in both cases) (Scheme 4). When the latter were subjected to Grubbs II catalyst in toluene at reflux temperature for 30 min, ring-closure to the macrocyclic olefins occurred in excellent yields (82% and 96%, respectively); in both cases the *E* isomer was obtained as the only isolable product. Treatment of the cyclization products with HF·pyridine then gave *E*-9,10-dehydro azathilones **5a** and **5b** in 67% and 51% yield, respectively, after purification by preparative HPLC. The reaction required careful monitoring by MS, in order to prevent cleavage of the urethane moiety, in particular in the case of the *tert*-butyl carbamate.

The conversion of **5a** into the saturated azathilone **3a** was performed with in situ generated diimide [31] under conditions that were established as the result of an extensive optimization process, involving the investigation of all four macrocyclic dienes prepared in the course of this study. Thus, the starting material **5a** was dissolved in CH_2_Cl_2_ together with a large excess of bis-potassium azodicarboxylate (PADA) and a solution of AcOH in CH_2_Cl_2_ was slowly added to the yellow suspension with a syringe pump. After 18 h the white suspension was filtered through a pad of silica and the filtrate was concentrated. The whole procedure was repeated until the conversion of starting material was deemed reasonable, in the case of **5a** the total reaction time amounted to 7 days. Azathilone **3a** was finally obtained in 66% yield after purification by preparative RP-HPLC. Similar conditions were used for the transformation of olefin **5b** into **3b** (for details see the Experimental Section); **3b** was obtained in 65% yield (after preparative RP-HPLC) together with 25% of unreacted starting material **5b**.

**Scheme 5:**
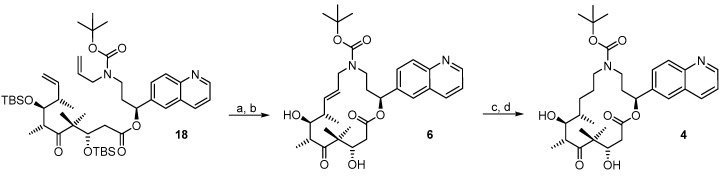
Synthesis of azathilones **6** and **4**. *Reagents and Conditions*: (**a**) EDC, DMAP, CH_2_Cl_2_, 0 °C → rt, 5 h, 90%; (**b**) Grubbs II (15 mol %), toluene, refl, 30 min, 81%; (**c**) HF·pyr, pyridine, THF, 0 °C → rt, 6 h, 55% (after HPLC purification); (**d**) PADA, MeOH/CH_2_Cl_2_, AcOH, rt, 77 h, 55% (after HPLC purification).

The elaboration of acid **16** and alcohol **15** into azathilones **6** and **4** was completely analogous to the synthesis of **5a/b** and **3a/b**, respectively (Scheme 5). The overall yield for the three step sequence from **15** to **6** was 41%; most notably, as for the cyclization of dienes **17a/b**, the RCM of the diene derived from **16** and **15**, i.e., **18**, provided the macrocyclic *E* olefin exclusively (82% yield).

The reduction of **6** was conducted in MeOH/CH_2_Cl_2_ under not yet optimized conditions (for details see the Experimental Section), to provide azathilone **4** in 55% yield (after preparative RP-HPLC) together with 18% of unreacted starting material **5b**. In contrast, the use of Crabtree’s catalyst in CH_2_Cl_2_ at 0 °C did not give any conversion of **5b**. Conversion was observed with Pt_2_O in EtOH at room temperature, but the reaction was non-selective and incomplete. Under these conditions **4** was obtained in only 14% yield and not entirely pure after tedious HPLC purification. Finally, the use of 2,4,6-triisopropylbenzenesulfonyl hydrazide and triethylamine in MeOH at room temperature [32] gave very slow conversion and the reaction was plagued by the formation of numerous side products, which necessitated extensive HPLC purification to provide **4** in 25% yield.

### 2.2. Biological Evaluation

**Figure 3:**
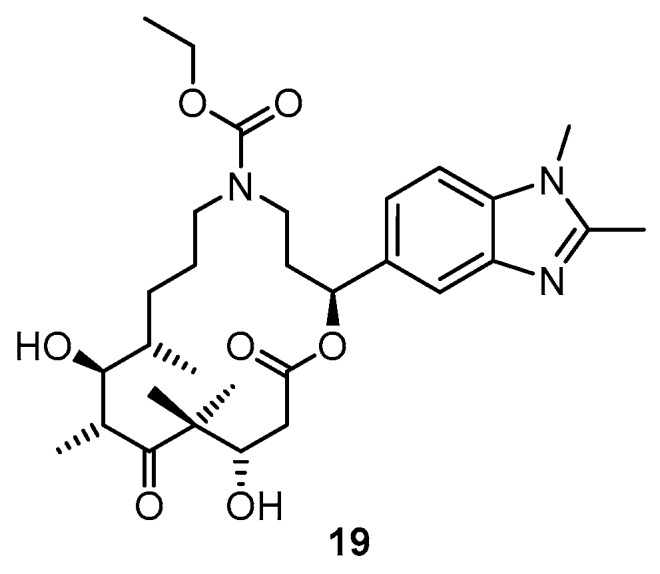
Molecular structure of azathilone **19**.

All azathilones prepared in this study were assessed for their in vitro antiproliferative activity on the human carcinoma cell lines A549 (lung) and MCF-7 (breast). As can be seen from the data compiled in Table 1, azathilones **3a** and **3b** are highly active against both cell lines, with IC_50_ values that are comparable with those for natural epothilone A. **3a** tends to be slightly more potent than **3b**, which is in line with the trend observed for azathilone **2** and the corresponding ethyl carbamate **19** (Figure 3), although the difference was more pronounced for the latter compound pair [16].

Importantly, azathilone **3a** is essentially equipotent with **2** on the A549 cell line, while **3b** is ca. 3-fold more potent than the corresponding dimethylbenzimidazole **19** [16]. These data show that also modified side chains other than the specific dimethylbenzimidazole moiety present in **2** and **19** can support highly potent antiproliferative activity in combination with the azathilone macrocycle.

As had been observed for **1** and **2** [16], desaturation of the C9-C10 bond in **3a** or **3b** to an *E* double bond results in a dramatic loss in cellular potency; the IC_50_ values for the corresponding dehydro analogs **5a** and **5b** are more than 1000-fold higher than those for the saturated parent compounds (Table 1). Although the number of examples of *E*-9,10-dehydroazathilones is still limited, collectively, the available data strongly suggest that the presence of a 9,10-*E* double bond is incompatible with potent bioactivity in the context of the azathilone structural framework. In contrast, the desaturation of the C9-C10 bond to an *E* double bond has proven to enhance (or at least not to diminish) cellular potency of natural epothilones or closely related polyketide-based epothilone analogs [14,26,27,33,34,35]. This seemed to suggests that the bioactive, tubulin-bound conformation of the azathilone macrocycle was likely to be different from that of the natural products. However, this hypothesis could not be validated in subsequent structural studies (vide infra) and the reasons for the divergent behavior of 9,10-dehyroazathilones and their polyketide-based congeners are still elusive at this point.

**Figure 4:**
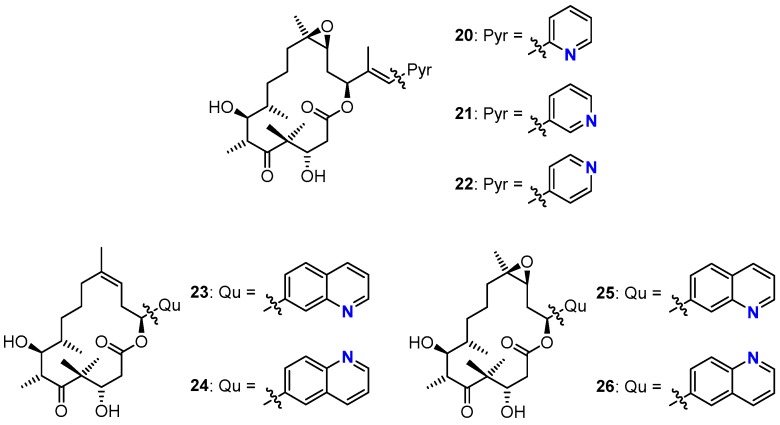
Molecular structures of previously reported pyridine- [36] and quinoline-based [25] epothilone B analogs.

In stark contrast to **3a**, its constitutional isomer **4** inhibits the proliferation of A549 and MCF-7 cells only with micromolar activity (IC_50_ values of 1034 nM and 998 nM, respectively, vs. 1.1 nM and 1.4 nM for **3a**, Table 1). Changes in the cellular activity of epothilone B or D analogs bearing pyridyl-vinyl or quinolyl side chains as a function of the position of the *N*-atom in the heterocycle have been observed previously, but the magnitude of the change is exceptionally high between azathilones **3a** and **4**. Thus, IC_50_ values of 0.3 nM, 4.3 nM, and 11.8 nM, respectively, have been reported for the three isomeric pyridyl-epothilone B analogs **20**–**22** (Figure 4) against the KB31 cell line (the only cell line for which data on these compounds have been reported) [36]. Likewise, a 110–140-fold difference in antiproliferative activity has been observed between isomeric quinolyl-epothilone D analogs **23** and **24** (Figure 4) (IC_50_ values of ca. 1 nM vs. ca. 100 nM); intriguingly, however, the corresponding epothilone B analogs **25** and **26** were found to be essentially equipotent (IC_50_ values < 1 nM for both compounds across three cell lines) [25].

Based on the available data it thus appears that the antiproliferative activity of azathilone-type epothilone analogs exhibits a significantly stronger dependence on the position of the N-atom in the side chain heterocycle than is the case for analogs with a natural macrolactone core structure. However, additional data are required to support (or disprove) the validity of this preliminary conclusion. Independent of this, although **4** is only a moderately potent cell growth inhibitor, it is still more active than its 9,10-dehydro variant **6** (Table 1), which re-enforces the above conclusions on the detrimental effects of the desaturation of the C9-C10 bond in azathilones even for a lower potency level of the saturated parent compound.

The differences in antiproliferative activity between **3a** and **5a** and also between **3a** and **4** are reflected in differences in the binding affinity of the compounds for stabilized, crosslinked microtubules (Table 2). Even if the correlation is not completely linear, it is clear from the data in Table 2 that *E*-9,10-dehydro azathilone **5a** and analog **4**, which has the *N*-atom in the heterocycle in the “non-natural” position, bind to microtubules with >10-fold lower affinity than **3a**.

Similar differences in microtubule-binding affinity have also been observed between **23** and **24** and between **25** and **26** (Figure 4), in spite of the fact that **25** and **26** display similar antiproliferative activity (vide supra). At the same time, the association constant for **3a** is ca. 10-fold lower than the ones reported for epothilone A or quinolyl-epothilone D (**23**), although the latter is a two orders of magnitude less potent growth inhibitor than **3a**. A fully consistent interpretation of these data is elusive at this point. While one could speculate that the potent antiproliferative activity of certain azathilones may result from additional interactions with cellular targets other than tubulin, at least for azathilone **2** we have previously shown that its overall biological profile is very similar to that of epothilone A [16].

A critical issue uncovered during the cellular profiling of azathilone **2** was a substantial loss in potency (>600-fold) against the Pgp-overexpressing multidrug-resistant cell line KB-8511 compared to the drug-sensitive parental KB31 line (IC_50_ values of 222 nM vs. 0.34 nM) [16]. The susceptibility of **2** to Pgp-mediated efflux has been confirmed in this study for the drug-sensitive/multidrug-resistant ovarian cancer cell line pair SK-OV-3/SKVLB1. As can be seen from Table 1, in analogy to the KB31/KB8511 system, azathilone **2** suffers from a dramatic drop in growth inhibitory activity in the Pgp-overexpressing, multidrug-resistant SKVLB1 cell line [37] in comparison with the sensitive SKOV3 line. An activity differential between SK-OV-3 and SKVLB1 cells is also obvious for azathilones **3a** and **3b**, although the corresponding resistance factors are clearly lower than for **2** (ca. 60 and 70, respectively, Table 1). A resistance factor of only 3 is observed for epothilone A. While these findings confirm that the replacement of the natural epothilone core structure by the azathilone macrocycle leads to inherently enhanced susceptibility to Pgp-mediated efflux, they also show that the magnitude of the effect depends on the specific nature of the side chain and may be further modulated (i.e., reduced) in new analogs. It should also be emphasized that both **3a** and **3b** are still potent growth inhibitors even in the SKVLB1 cell line, even if their IC_50_ values are no longer in the single digit nanomolar range.

In order to gain insight into the interactions between azathilones and the tubulin/microtubule system at the molecular level, we have investigated the conformational properties of azathilone **2**, both in its free state in aqueous solution and when bound to tubulin, by means of NMR spectroscopy and computational methods.

### 2.3. Structural Studies

The conformation of **2** free in aqueous solution was determined by solution NMR experiments in D_2_O at 500 MHz and 298 K. The complete assignment of the ^1^H-NMR resonances of **2** was achieved on the basis of 1D, 2D TOCSY and 2D ROESY experiments. Due to the intermediate size of the compound, which leads to near-zero longitudinal NOE effects, the conformational information in the free state was derived from 2D ROESY experiments. For this purpose, an ensemble of 250 structures was obtained from a restrained-free Monte Carlo conformational search, as implemented in Macromodel software. The combined computational and NMR data indicated the presence of two families of low energy conformers of **2** in aqueous solution, with very similar conformations of the macrocycle, but distinct torsion angles about the C15-C16 bond (for atom numbering see Figure 1). In fact, ROESY cross peaks of approximately equal intensity were observed between H15 and H17 and between H15 and H27, respectively. This finding is indicative of rapid rotation around the C15-C16 bond on the NMR time scale, which suggests that **2** in aqueous solution is present in an equilibrium between two equally populated conformers A (C14-C15-C16-C17 dihedral angle of ca. 170°) and B (C14-C15-C16-C17 dihedral angle of ca. 10°) (Figure 1). In both cases, the conformation of the macrocycle was found to be similar to that reported for epothilone A free in aqueous solution [40] (Table S1) and also to that of tubulin-bound **2** (vide infra).

**Figure 5:**
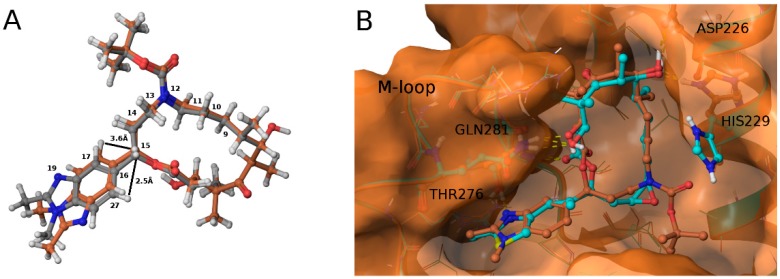
Tubulin-bound conformation of azathilone **2** and 3D model of its interaction with β-tubulin. (**A**) Overlay of the two conformers A (**orange**) and B (**grey**) of tubulin-bound azathilone **2** as derived from the TR-NOESY data; (**B**) 3D model of the azathilone **2**/β-tubulin complex (**2** in orange, conformer B) as derived from TR-NOE and STD NMR experiments, docking, and CORCEMA-ST calculations and based on the structure of β-tubulin in complex with epothilone A [45] (for details see text). Epothilone A is shown in blue.

As a next step, we undertook the determination of the bioactive, tubulin-bound conformation of azathilone **2** by means of transferred nuclear Overhauser enhancement (TR-NOE) measurements. Strong negative TR-NOE cross-peaks were observed for azathilone **2** in the presence of tubulin under conditions where α/β tubulin heterodimers have been shown not to assemble into microtubule polymers [41]. The NOE-derived distances in the bound state are summarized in Table S2 and the corresponding conformations are depicted in Figure 5A. No significant differences were found between the conformation of the macrocyclic core structure of **2** in the tubulin-bound state and free in solution, thus indicating that the macrocycle in the free state is pre-organized for binding. In addition, the analysis of the intensities of the cross peaks H15–H17 and H15–H27 revealed that the dimethylbenzimidazole side chain adopts two distinct orientations also in the bound state (Figure 5A); in contrast to the near balanced conformational equilibrium in solution, however, conformer A is clearly more populated than B.

We have previously reported similar observations for the side chain conformations of tubulin-bound epothilone A or B [41], where a *syn*-periplanar conformation about the C17-C18 bond (C16-C17-C18-C19 torsion angles of −28° (epothilone A) and −29° (epothilone B) is substantially more populated (>80%) than the *anti*-periplanar arrangement. This conformation places the heterocyclic nitrogen atom in the thiazole ring in epothilones A and B approximately in the same position (relative to the macrocycle) as N^19^ in conformer B in azathilone **2** (Figure 5B) (for atom numbering see Figure 1). Virtually the same side chain orientation as in tubulin-bound **2** has also been observed for quinoline-based epothilone analog **25** (Figure 4) [42]. In analogy to the free state in solution, the conformation of the macrocycle in tubulin-bound **2** is overall similar to that in tubulin-bound epothilone A or B [41,43] (Figure 5B and Table S3); deviations do exist for individual torsion angles, but mainly for those bonds including, or being adjacent to N^12^. Of particular interest is the torsion angle about the C9-C10 bond, which was determined to be around −150° (Table S3). However, this number should be interpreted with caution, as the accuracy of the data in this region of the structure is limited, due to signal overlap in the NMR spectra. It thus remains unclear at this point if the profound loss in biological activity incurred upon desaturation of the C9-C10 bond in azathilones could be caused by a simple incompatibility of the bioactive conformation with a fixed C9-C10 torsion angle of 180° or if other (additional) effects come into play. In this context it should be remembered that the introduction of a double bond will affect the (intrinsically) preferred torsion angles about adjacent bonds due to 1,3 allylic strain effects [44]. Interestingly, such secondary effects do not seem to be of major relevance for natural epothilones and closely related analogs (vide supra), where the conformation about the C9-C10 bond in the tubulin-bound state has been clearly established to be close to *anti*-periplanar [41,42,43].

Finally, we have performed saturation transfer (STD)-NMR experiments, in order to determine the major interaction sites in **2** with the tubulin α/β heterodimer and to construct a model for bound **2** in the luminal epothilone binding site on β-tubulin. STD-NMR is based on the magnetization transfer from a given protein to protons of a bound ligand [46]. Only bound ligands show STD signals, which are strongest for those ligand protons that are in closest contact with the protein. Thus, the intensity of STD signals for individual ligand protons reflects their proximity to the protein surface. Clear STD signals were detected for azathilone **2** in the presence of α/β tubulin heterodimer (for a ca. 15:1 molar ratio of **2** vs. tubulin). The STD intensities for individual protons are plotted for both conformers A and B in Figure S2. In order to derive a model for the complex between **2** and tubulin, the compound was then modelled into the luminal epothilone binding site on β-tubulin (PDB code 4I50) with AutoDock Vina [47]. In view of the similar tubulin-bound conformations of **2** and epothilone A, a rigid docking protocol was employed initially, based on the tubulin-bound conformation of **2** that had been derived from the TR-NOE data. In a second step, the obtained binding poses were then further refined. Subsequently, CORCEMA-ST calculations [48] were performed with the best binding poses obtained in the docking calculations to determine which of the proposed binding modes provided the best agreement with the STD experimental data. Different torsion angles were considered for both the C15-C16 bond and the *O*-C(CH_3_)_3_ bond in the *tert-*butoxycarbonyl moiety attached to N12. It is worth noting that the calculated STD profiles obtained for conformers A and B are very similar (Figure S2) and both are in good agreement with the experimental data. This clearly indicates that both conformers represent feasible solutions for the tubulin-bound structure of **2**.

**Figure 6:**
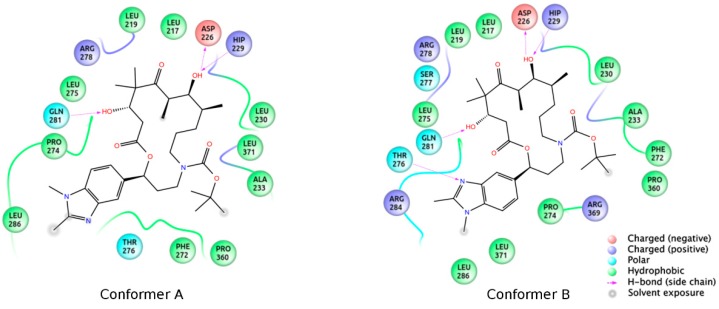
Key intermolecular contacts between tubulin and azathilone **2**.

As illustrated in Figure 5B, the predicted overall binding mode of **2** to β-tubulin is very similar to that of epothilone A for both conformers A and B. Key contacts between **2** and tubulin are depicted in Figure 6 and include a mix of hydrophobic and polar interactions.

Of particular note is the fact that polar interactions between C3OH and the side chain of Gln 281, C7OH and the side chain carboxylate group of Asp226, and N19 (corresponding to the thiazole N in epothilone A) and the side chain hydroxyl group of Thr 276 (for conformer B) are also observed in the crystal structure of the tubulin-epothilone A complex [45], thus reflecting the similarities in the binding poses of tubulin-bound **2** and natural epothilones.

In summary, we have used ring-closing olefin metathesis to prepare a set of new 12-aza-epothilones (azathilones) incorporating quinoline side chains and bearing different N12-substituents. Quinoline-based azathilones with the side chain *N*-atom in the *meta*-position to the C15 atom in the macrocycle were found to bind to microtubules with high affinity and to be potent inhibitors of cancer cell growth in vitro. In contrast, analogs with the quinoline nitrogen in the position *para* to C15 were ca. 1000-fold less active in cells. Collectively, the data illustrate that the benzimidazole side chain of the previously described azathilone **2** is not a specific requirement for potent growth inhibitory activity. We have determined the conformation of azathilone **2** bound to tubulin heterodimers based on TR-NOE measurements and a model for the posture of the compound in its binding site on β-tubulin was deduced through a combination of STD measurements and CORCEMA-ST calculations. The tubulin-bound structure of **2** was found to be overall similar to that of epothilones A and B. The current structural data, thus, do not offer a rationale for the substantial loss in antiproliferative activity incurred upon desaturation of the C9-C10 bond in the azathilone macrocycle to an *E* double, a phenomenon that is not observed for natural epothilones.

## 3. Experimental Section

### 3.1. General Information

All solvents used for reactions were purchased as anhydrous grade from Sigma-Aldrich (puriss.; dried over molecular sieves; H_2_O <0.005%) (Buchs, Switzerland) and used without further purification. Solvents for extractions, flash column chromatography (FC) and thin layer chromatography (TLC) were purchased as commercial grade and distilled prior to use. Commercially available starting materials and reagents were also from Sigma-Aldrich and used without further purification, unless otherwise noted. In general, reactions were magnetically stirred and monitored by TLC performed on Merck TLC aluminum sheets (silica gel 60 F_254_) (Merck, Darmstadt, Germany). Spots were visualized with UV light (λ = 254 nm) or through staining with Ce_2_(SO_4_)_3_/phosphomolybdic acid/H_2_SO_4_ or KMnO_4_/K_2_CO_3_. Chromatographic purification of products by FC was performed using Sigma-Aldrich silica gel 60 for preparative column chromatography (particle size 40–63 µm). ^1^H- and ^13^C-NMR spectra were recorded in CDCl_3_ (unless otherwise noted) on a Bruker AV-400 400 or a Bruker AV-500 500 MHz spectrometer (Bruker, Karlsruhe, Germany) at room temperature. Chemical shifts (δ) are referenced to the solvent signal as an internal standard (chloroform δ 7.26 ppm for ^1^H and δ 77.00 ppm for ^13^C spectra; DMSO-*d*_6_ δ 2.50 ppm for ^1^H and δ 39.43 ppm for ^13^C spectra). All ^13^C-NMR spectra were measured with complete proton decoupling. Data for NMR spectra are reported as follows: s = singlet, d = doublet, t = triplet, q = quartet, quint. = quintet, sext. = sextet, m = multiplet, br = broad signal, J = coupling constant in Hz. Infrared spectra (IR) were recorded on a Jasco FT/IR-6200 instrument (Jasco Switzerland, Brechbühler AG, Schlieren, Switzerland). Resonance frequencies are given as wavenumbers in cm^−1^. Optical rotations were measured on a Jasco P-1020 polarimeter (Jasco Switzerland, Brechbühler AG) operating at the sodium D line with a 10 mm or 100 mm path length cell and are reported as follows: ${\left[a\right]}_{D}^{T}$, concentration (g/100 mL), and solvent. HRMS (ESI) spectra were obtained on a Varian IonSpec system (Agilent Technologies (Schweiz), Basel, Switzerland). For analytical high-performance liquid chromatography (HPLC) the following combination of devices by VWR HITACHI (VWR International AG, Dietikon, Switzerland) was used: Diode array detector L-2455, autosampler L-2200, pump L-2130. For preparative HPLC a device by Gilson (Gilson (Schweiz) AG, Mettmenstetten, Switzerland) was used.

### 3.2. Chemistry

*(S)-N-(3-(t-Butyldimethylsilyloxy)-3-(quinolin-7-yl)propyl) prop-2-en-1-amine* (**9**). To heat-activated molecular sieves (4 Å, 650 mg) was added a solution of aldehyde **8** [25] (522 mg, 1.65 mmol) in 8 mL THF. To this solution were added 0.6 mL of allylamine (7.86 mmol) and the mixture was heated to 50 °C for 24 h. It was then filtered through a pad of dry Celite™, the residue was washed with THF and the combined filtrates were concentrated under reduced pressure to give a yellow oil. This material showed the expected mass for the imine and was directly used as such. For the reduction, 67 mg of NaBH_4_ (1.7 mmol) were placed in a 10 mL two-necked flask at 0 °C and a solution of the crude imine in 3 mL MeOH was added (gas formation could be observed). After 20 min the reaction mixture was diluted with water and extracted with EtOAc. The combined organic fractions were washed with brine, dried over MgSO_4_ and concentrated in vacuo. Purification of the residue by FC (hexane/EtOAc 4:1 → 1:1 + 1% Et_3_N) furnished 445 mg (76%) of the desired amine **9** as a slightly yellow oil; ${\left[\alpha \right]}_{D}^{RT}$ = −54.0° (*c* = 1.37, CH_2_Cl_2_). ^1^H-NMR (400 MHz, CDCl_3_): δ = 8.87 (dd, *J* = 4.3 Hz, 1.7 Hz, 1H), 8.09 (dd, *J* = 8.3 Hz, 1.2 Hz, 1H), 7.94 (br s, 1H), 7.75 (d, *J* = 8.4 Hz, 1H), 7.54 (dd, *J* = 8.4 Hz, 1.7 Hz, 1H), 7.33 (dd, *J* = 8.2 Hz, 4.2 Hz, 1H), 5.85 (m, 1H), 5.10 (dq, *J* = 17.2 Hz, 1.6 Hz, 1H), 5.02 (dq, *J* = 10.3 Hz, 1.4 Hz, 1H), 4.97 (dd, *J* = 7.3 Hz, 4.8 Hz, 1H), 3.17 (dt, *J* = 6.1 Hz, 1.3 Hz, 2H), 2.66 (t, *J* = 7.0 Hz, 2H), 2.01–1.84 (br m, 2H), 0.87 (s, 9H), 0.03 (s, 3H), −0.17 (s, 3H); NH not visible. ^13^C-NMR (100 MHz, CDCl_3_): δ = 150.5, 148.2, 147.0, 136.9, 135.8, 127.7, 127.4, 125.7, 124.9, 120.7, 115.7, 73.5, 52.5, 45.8, 40.6, 25.8 (3 × CH_3_), 18.1, −4.6, −5.0. HRMS (ESIpos): calcd. for C_21_H_33_N_2_OSi [M + H]^+^: 357.2357, found: 357.2361.

*(S)-t-Butyl allyl(3-(t-butyldimethylsilyloxy)-3-(quinolin-7-yl)propyl)carbamate* (**9A**). A solution of amine **9** (416 mg, 1.17 mmol) and Boc_2_O (390 mg, 1.75 mmol) was stirred at rt for 14 h. Ethanolamine (1 mL) was then added and the mixture was stirred for one additional hour at rt and then concentrated in vacuo. To the resulting crude product water was added and the mixture was extracted with Et_2_O and CH_2_Cl_2_. The combined organic layers were dried over MgSO_4_ and concentrated in vacuo. Purification of the residue by FC (hexane/EtOAc 10:1 → 1:1 + 1% Et_3_N) gave 460 mg (86%) of the desired carbamate **9A** as colorless oil; R_f_ = 0.8 (hexane/EtOAc 1:1). ${\left[\alpha \right]}_{D}^{RT}$ = −36.2° (*c* = 1.50, CH_2_Cl_2_). ^1^H-NMR (400 MHz, CDCl_3_): δ = 8.84 (dd, *J* = 4.1 Hz, 1.5 Hz, 1H), 8.06 (d, *J* = 7.9 Hz, 1H), 7.94 (s, 1H), 7.72 (d, *J* = 8.4 Hz, 1H), 7.50 (dd, *J* = 8.5 Hz, 1.2 Hz, 1H), 7.29 (dd, *J* = 8.1 Hz, 4.2 Hz, 1H), 5.67 (m, 1H), 5.01 (m, 1H), 4.97 (dq, *J* = 7.7 Hz, 1.4 Hz, 1H), 4.84 (dd, *J* = 6.6 Hz, 4.7 Hz, 1H), 3.72 (m, 2H), 3.21 (m, 2H), 1.95 (m, 2H), 1.33 (s, 9H), 0.85 (s, 9H), 0.01 (s, 3H), −0.20 (s, 3H). ^13^C-NMR (100 MHz, CDCl_3_): δ = 155.2, 150.4, 148.1, 146.5, 135.7, 134.1, 127.7, 127.4, 125.6, 124.7, 120.7, 116.3, 79.2, 73.0, 49.6, 43.6, 38.7, 28.3 (3 × CH_3_), 25.7 (3 × CH_3_), 18.0, −4.7, −5.1. HRMS (ESIpos): calcd. for C_26_H_40_N_2_O_3_SiNa [M + Na]^+^: 479.27004, found: 479.27007.

*(S)-t-Butyl allyl(3-hydroxy-3-(quinolin-7-yl)propyl)carbamate* (**10**). To a solution of silyl-ether **9A** (200 mg, 0.44 mmol) in 5 mL THF were added 1.32 mL (1.32 mmol) of a TBAF-solution (1 M in THF) at rt and the mixture was stirred for 5 h. The reaction was quenched with saturated, aqueous NH_4_Cl solution and the mixture was extracted with EtOAc. The combined organic layers were washed with H_2_O, dried over MgSO_4_ and concentrated in vacuo. The residue was purified by FC (hexane/EtOAc 2:1 → 1:1) to give 152 mg (quantitative yield) of the desired free alcohol **10** as a colorless oil; R_f_ = 0.1 (hexane/EtOAc 2:1). ${\left[\alpha \right]}_{D}^{RT}$ = −1.94° (*c* = 1.05, CH_2_Cl_2_). ^1^H-NMR (400 MHz, CDCl_3_): δ = 8.85 (dd, *J* = 4.2 Hz, 1.4 Hz, 1H), 8.09 (dm, *J* = 8.1 Hz, 1H), 8.01 (br s, 1H), 7.76 (d, *J* = 8.5 Hz, 1H), 7.62 (m, 1H), 7.33 (dd, *J* = 8.1 Hz, 4.2 Hz, 1H), 5.78 (m, 1H), 5.14 (br s, 1H), 5.10 (d, *J* = 4.7 Hz, 1H), 4.82 (m, 1H), 4.72 (m, 1H, OH), 3.88 (dd, *J* = 15.7 Hz, 5.4 Hz, 2H), 3.72 (m, 1H), 3.12 (m, 1H), 2.02 (m, 1H), 1.81 (m, 1H), 1.44 (s, 9H). ^13^C-NMR (100 MHz, CDCl_3_): δ = 171.1, 150.4, 148.2, 146.0, 135.8, 133.9, 127.9, 127.4, 125.6, 124.8, 120.8, 116.7, 80.4, 69.9, 50.1, 43.4, 37.9, 28.3 (3 × CH_3_). HRMS (ESIpos): calcd. for C_20_H_26_N_2_O_3_SiNa [M + Na]^+^: 365.1836, found: 365.1834.

*(S)-Ethyl allyl(3-(t-butyldimethylsilyloxy)-3-(quinolin-7-yl) propyl)carbamate* (**9B**). To a solution of amine **9** (150 mg, 0.42 mmol) and K_2_CO_3_ (176 mg, 1.26 mmol) in 4 mL acetone was added a solution of ethyl chloroformate (70 mg, 0.63 mmol) in 1 mL acetone at 0 °C. The mixture was stirred at rt and the reaction was quenched with water. The aqueous layer was extracted with EtOAc and the combined organic layers were dried over MgSO_4_ and concentrated in vacuo. Purification of the residue by FC (hexane/EtOAc 2:1) gave 156 mg (87%) of the desired carbamate **9B** as a yellow oil; R_f_ = 0.2 (hexane/EtOAc 4:1); 0.4 (hexane/EtOAc 2:1). ${\left[\alpha \right]}_{D}^{RT}$ = −41.8° (*c* = 1.79, CH_2_Cl_2_). ^1^H-NMR (400 MHz, CDCl_3_): δ = 8.88 (dd, *J* = 4.3 Hz, 1.6 Hz, 1H), 8.10 (d, *J* = 8.2 Hz, 1H), 7.95 (br s, 1H), 7.76 (d, *J* = 8.4 Hz, 1H), 7.53 (dd, *J* = 8.4 Hz, 1.6 Hz, 1H), 7.35 (dd, *J* = 8.3 Hz, 4.3 Hz, 1H), 5.69 (m, 1H), 5.03 (m, *2*H), 4.88 (dd, *J* = 7.1 Hz, 5.9 Hz, 1H), 4.06 (q, *J* = 7.2 Hz, 2H), 3.78 (m, 2H), 3.26 (m, 2H), 1.98 (m, 2H), 1.16 (t, *J* = 7.1 Hz, 3H), 0.89 (s, 9H), 0.05 (s, 3H), −0.17 (s, 3H). ^13^C-NMR (100 MHz, CDCl_3_): δ = 156.2, 150.6, 148.2, 146.4, 135.8, 133.9, 127.8, 127.5, 125.8, 124.8, 120.8, 116.8, 72.9, 61.1, 49.7, 43.6, 38.6, 25.8 (3 × CH_3_), 18.1, 14.6, −4.6, −5.1. IR (film): ῦ = 2931, 2857, 1698, 1472, 1417, 1384, 1250, 1092, 836, 776, 677. HRMS (ESIpos): calcd. for C_24_H_36_N_2_O_3_SiNa [M + Na]^+^: 451.2387, found: 451.2385.

*(S)-Ethyl allyl(3-hydroxy-3-(quinolin-7-yl)propyl)carbamate* (**11**). To a solution of silyl-ether **9B** (154 mg, 0.36 mmol) in 4 mL THF were added 1.08 mL (1.08 mmol) TBAF-solution (1M in THF) at rt. The mixture was stirred at rt for 7 h; aqueous NH_4_Cl solution was then added and the solution was extracted with EtOAc. The combined organic layers were washed with H_2_O, dried over MgSO_4_ and concentrated in vacuo. The residue was purified by FC (hexane/EtOAc 2:1 → 1:1) to furnish 109 mg (96%) of the free alcohol **11** as a yellow oil; R_f_ = 0.1 (hexane/EtOAc 2:1). ${\left[\alpha \right]}_{D}^{RT}$ = −8.57° (*c* = 1.25, CH_2_Cl_2_). ^1^H-NMR (400 MHz, CDCl_3_): δ = 8.86 (dd, *J* = 4.3 Hz, 1.7 Hz, 1H), 8.10 (dd, *J* = 8.3 Hz, 1.0 Hz, 1H), 8.01 (br s, 1H), 7.77 (d, *J* = 8.5 Hz, 1H), 7.62 (d, *J* = 6.2 Hz, 1H), 7.34 (dd, *J* = 8.4 Hz, 4.3 Hz, 1H), 5.78 (m, 1H), 5.15 (br s, 1H), 5.11 (m, 1H), 4.85 (d, *J* = 8.6 Hz, 1H), 4.15 (q, *J* = 7.1 Hz, 2H), 3.95–3.73 (m, 3H), 3.19 (m, 1H), 2.05 (m, 1H), 1.86 (m, 1H), 1.23 (t, *J* = 7.1 Hz, 3H); OH-proton not visible. ^13^C-NMR (100 MHz, CDCl_3_): δ = 157.6, 150.5, 148.2, 145.9, 135.8, 133.5, 127.9, 127.4, 125.6, 124.8, 120.8, 117.0, 70.0, 61.9, 49.7, 43.6, 37.7, 14.6. IR (film): ῦ = 3392 (br), 2980, 2926, 1673, 1472, 1415, 1241, 1069, 839, 771, 677 cm^−1^. HRMS (ESIpos): calcd. for C_18_H_22_N_2_O_3_Na [M + Na]^+^: 337.1523, found: 337.1520.

*(S)-3-((t-Butyldimethylsilyl)oxy)-1-((3aS,6R,7aR)-8,8-dimethyl-2,2-dioxidotetrahydro-3H-3a,6-methano-benzo[c]isothiazol-1(4H)-yl)-3-(quinolin-6-yl)propan-1-one* (**12A**). To a solution of 4.53 g (10.90 mmol) of (*S*)-1-((3a*S*,6*R*,7a*R*)-8,8-dimethyl-2,2-dioxidotetrahydro-3*H*-3a,6-methanobenzo[c]isothiazol-1(4*H*)-yl)-3-hydroxy-3-(quinolin-6-yl)propan-1-one (3:1 mixture of diastereoisomers) [25] in 75 mL of DMF were added 2.25 g (32.9 mmol) of imidazole and 2.52 g (16.4 mmol) of TBSCl and the reaction mixture was stirred at 40 °C for 16 h. The solution was then evaporated, CH_2_Cl_2_ and water were added, and the organic layer was separated. The aqueous solution was additionally extracted with CH_2_Cl_2_ and the combined organic extracts were dried over MgSO_4_, and evaporated. The residue was purified by FC (hexane/EtOAc 3:2, three runs) to give 3.76 g (65%) of protected alcohol **12A** as a light-yellow solid (single isomer) and 1.15 g (20%) of the corresponding 3*R*-isomer; R_f_ = 0.7 (hexane/EtOAc 1:1). ^1^H-NMR (400 MHz, CDCl_3_): δ = 8.86 (dd, *J* = 4.3 Hz, 1H), 8.10 (dm, *J* = 8.3 Hz, 1H), 8.03–8.01 (m, 1H), 7.75 (d, *J* = 7.0 Hz, 1H), 7.75 (d, *J* = 7.5 Hz, 1H), 7.35 (dd, *J* = 8.3 Hz, 4.2 Hz, 1H), 5.39 (t, *J* = 6.8 Hz, 1H), 3.73 (d, *J* = 7.7 Hz, 1H), 3.33 (s, 2H), 3.22 (dd, *J* = 14.9 Hz, 6.9 Hz, 1H), 3.14 (dd, *J* = 14.9 Hz, 6.9 Hz, 1H), 1.92 (dd, *J* = 13.6 Hz, 7.9 Hz, 1H), 1.83–1.64 (br, m, 3H), 1.62 (m, 1H), 1.26 (m, 2H), 0.84 (s, 9H), 0.78 (s, 3H), 0.45 (s, 3H), 0.05 (s, 3H), −0.17 (s, 3H). ^13^C-NMR (100 MHz, CDCl_3_): δ = 168.9, 150.2, 148.1, 141.8, 136.1, 129.5, 128.3, 127.9, 125.0, 121.1, 71.9, 64.9, 52.9, 48.1, 47.5, 46.5, 44.5, 38.2, 32.8, 26.3, 25.7 (3 × CH_3_), 20.0, 19.6, 18.1, −4.7, −5.0.

*(S)-3-((t-Butyldimethylsilyl)oxy)-3-(quinolin-6-yl)propanal* (**13**). To a solution of 3.76 g (7.11 mmol) of TBS-ether **12A** in 35 mL of CH_2_Cl_2_ were added dropwise 18 mL of a 1M solution of DIBAL-H (18 mmol) at −78 °C under Ar over a period of 30 min. The mixture was then stirred at −78 °C for 6 h. Water was then added to quench the reaction, the mixture was diluted with additional water (200 mL), CH_2_Cl_2_ (200 mL) and 45 mL of 1 N NaOH. The organic layer was then separated and the aqueous solution was additionally extracted with CH_2_Cl_2_ (2 times). The combined organic extracts were washed with water, dried over MgSO_4_, and the solvent was evaporated. Purification of the residue by FC with hexane/EtOAc 1/1 (3 runs) gave 2.29 g (102%) of slightly impure aldehyde **13** as a yellow oil; R_f_ = 0.6 (hexane/EtOAc 1:1). ${\left[\alpha \right]}_{D}^{RT}$ = −76.2° (c = 1.09 in CH_2_Cl_2_). ^1^H-NMR (400 MHz, CDCl_3_): δ = 9.77 (dd, *J* = 2.4 Hz, *J* = 2.0 Hz, 1H), 8.86 (dd, *J* = 4.3 Hz, 1.8 Hz, 1H), 8.10 (dm, *J* = 8.4 Hz, 1H), 8.06 (d, *J* = 8.7 Hz, 1H), 7.73 (m, 1H), 7.68 (dd, *J* = 8.7 Hz, 1.9 Hz, 1H), 7.36 (dd, *J* = 8.4 Hz, *J* = 4.2 Hz, 1H), 5.38 (dd, *J* = 8.0 Hz, *J* = 4.2 Hz, 1H), 2.91 (ddd, *J* = 15.9 Hz, J = 8 Hz, *J* = 2.5 Hz, 1H), 2.68 (ddd, *J* = 16.0 Hz, *J* = 4.3 Hz, *J* = 1.9 Hz, 1H), 0.84 (s, 9H), 0.03 (s, 3H), −0.17 (s, 3H). ^13^C-NMR (100 MHz, CDCl_3_): δ = 200.6, 150.4, 147.9, 142.0, 136.0, 129.9, 127.9, 127.3, 124.1, 121.4, 70.3, 53.8, 25.6 (3 × CH_3_), 18.0, −4.7, −5.2. HRMS (ESIpos): calcd. for C_18_H_25_NO_2_SiNa [M + Na]^+^: 338.1547, found: 338.1539.

*(S)-N-(3-(t-Butyldimethylsilyloxy)-3-(quinolin-6-yl)propyl)prop-2-en-1-amine* (**14**). To heat-activated molecular sieves (4 Å, 600 mg) was added a solution of aldehyde **13** (250 mg, 0.79 mmol) in 5 mL THF. To this solution were added 0.3 mL of allylamine (3.93 mmol) and the mixture was heated to 50 °C for 24 h. It was then filtered through a pad of dry Celite™, the residue was washed with THF and the combined filtrates were concentrated under reduced pressure to give a yellow oil. For the reduction, 32 mg of NaBH_4_ (0.81 mmol) were placed in a 10 mL two-necked flask at 0 °C and a solution of the crude imine in 3 mL MeOH was added (gas formation could be observed). After 20 min the reaction mixture was diluted with water and extracted with EtOAc. The combined organic extracts were washed with brine, dried over MgSO_4_ and concentrated in vacuo. Purification of the residue by FC (hexane/EtOAc 4:1 → 1:1 + 1% Et_3_N) furnished 188 mg (67%) of the desired amine **14** as slightly yellow oil; ${\left[\alpha \right]}_{D}^{RT}$ = −58.1° (*c* = 1.29, CH_2_Cl_2_). ^1^H-NMR (400 MHz, CDCl_3_): δ = 8.82 (dd, *J* = 4.3 Hz, 1.8 Hz, 1H), 8.06 (dd, *J* = 8.4 Hz, 1.4 Hz, 1H), 8.02–8.00 (m, 1H), 7.65 (dd, *J* = 7.1 Hz, 2.1 Hz, 2H), 7.31 (dd, *J* = 8.3 Hz, 4.3 Hz, 1H), 5.82 (m, 1H), 5.07 (dq, *J* = 17.2 Hz, 1.6 Hz, 1H), 5.00 (dq, *J* = 10.3 Hz, 1.6 Hz, 1H), 4.93 (dd, *J* = 7.5 Hz, 4.7 Hz, 1H), 3.15 (dt, *J* = 6.0 Hz, 1.2 Hz, 2H), 2.63 (dd, *J* = 7.3 Hz, 6.7 Hz, 2H), 1.97–1.80 (br m, 2H), 0.85 (s, 9H), 0.01 (s, 3H), −0.21 (s, 3H); NH proton not visible. ^13^C-NMR (100 MHz, CDCl_3_): δ = 149.9, 147.7, 143.7, 136.8, 135.8, 129.3, 127.8, 127.8, 123.8, 121.0, 115.6, 73.3, 52.5, 45.7, 40.7, 25.7 (3 × CH_3_), 18.1, −4.7, −5.1. HRMS (ESIpos): calcd. for C_21_H_32_N_2_OSiNa [M + Na]^+^: 379.2176, found: 379.2178.

*(S)-t-Butyl allyl(3-(t-butyldimethylsilyloxy)-3-(quinolin-6-yl)propyl)carbamate* (**14A**). A solution of amine **14** (163 mg, 0.46 mmol) and Boc_2_O (154 mg, 0.69 mmol) in 7 mL CH_2_Cl_2_ was stirred at rt for 14 h. 1 mL of ethanolamine was then added and stirring was continued for one additional hour. The mixture was concentrated in vacuo, water was added to the residue, and the mixture was extracted with Et_2_O and CH_2_Cl_2_. The combined organic layers were dried over MgSO_4_ and concentrated in vacuo. Purification of the residue by FC (hexane/EtOAc 10:1 → 4:1 + 1% Et_3_N) gave 180 mg (86%) of the desired carbamate **14B** as a colorless oil; R_f_ = 0.6 (hexane/EtOAc 2:1). ${\left[\alpha \right]}_{D}^{RT}$ = −37.5° (*c* = 1.29, CH_2_Cl_2_). ^1^H-NMR (400 MHz, CDCl_3_): δ = 8.83 (dd, *J* = 4.0 Hz, 1.6 Hz, 1H), 8.08 (dm, *J* = 8.2 Hz, 1H), 8.03 (d, *J* = 9.1 Hz, 1H), 7.67–7.64 (m, 2H), 7.33 (dd, *J* = 8.3 Hz, 4.2 Hz, 1H), 5.68 (m, 1H), 5.02 (q, *J* = 1.2 Hz, 1H), 4.99 (dq, *J* = 9.2 Hz, 1.5 Hz, 1H), 4.83 (dd, *J* = 7.0 Hz, 4.8 Hz, 1H), 3.72 (m, 2H), 3.20 (m, 2H), 1.95 (m, 2H), 1.34 (s, 9H), 0.86 (s, 9H), 0.01 (s, 3H), −0.20 (s, 3H). ^13^C-NMR (100 MHz, CDCl_3_): δ = 155.3, 150.0, 147.7, 143.4, 135.9, 134.1, 129.4, 127.9, 127.6, 123.9, 121.1, 116.3, 79.3, 72.9, 49.7, 43.6, 38.8, 28.3 (3 × CH_3_), 25.7 (3 × CH_3_), 18.1, −4.7, −5.1. HRMS (ESIpos): calcd. for C_26_H_40_N_2_O_3_SiNa [M + Na]^+^: 479.27004, found: 479.2699.

*(S)-t-Butyl allyl*[3-hydroxy-3-(quinolin-6-yl)propyl] *carbamate* (**15**). To a solution of silyl-ether **14B** (200 mg, 0.44 mmol) in 5 mL THF were added 1.32 mL (1.32 mmol) TBAF-solution (1 M in THF) and the mixture was stirred at rt for 5 h. The reaction was quenched with saturated aqueous NH_4_Cl solution and the mixture was extracted with EtOAc. The combined organic layers were washed with H_2_O, dried over MgSO_4_ and concentrated in vacuo. The residue was purified by FC (hexane/EtOAc 2:1 → 1:1) to furnish 143 mg (95%) of the desired free alcohol **15** as a colorless oil; R_f_ = 0.2 (hexane/EtOAc 2:1).${\left[\alpha \right]}_{D}^{RT}$ = −9.49° (*c* = 1.13, CH_2_Cl_2_). ^1^H-NMR (400 MHz, CDCl_3_): δ = 8.85 (dd, *J* = 4.2 Hz, 1.5 Hz, 1H), 8.12 (dd, *J* = 8.4 Hz, 1.2 Hz, 1H), 8.04 (d, *J* = 8.7 Hz, 1H), 7.84 (br s, 1H), 7.66 (dd, *J* = 8.8 Hz, 2.0 Hz, 1H), 7.36 (dd, *J* = 8.2 Hz, 4.2 Hz, 1H), 5.80 (m, 1H), 5.16 (br s, 1H), 5.12 (d, *J* = 6.3 Hz, 1H), 4.80 (m, 2H; 1xOH), 3.91 (dd, *J* = 15.7 Hz, 5.4 Hz, 2H), 3.72 (m, 1H), 3.10 (m, 1H), 2.03 (m, 1H), 1.76 (m, 1H), 1.45 (s, 9H). ^13^C-NMR (100 MHz, CDCl_3_): δ = 171.13, 150.08, 147.71, 142.60, 136.07, 133.83, 129.35, 128.15, 127.72, 123.79, 121.18, 116.75, 80.53, 69.80, 50.12, 43.33, 38.09, 28.36 (3 × CH_3_). HRMS (ESIpos): calcd. for C_20_H_26_N_2_O_3_SiNa [M + Na]^+^: 365.1836, found: 365.1831.

*(3S,6R,7S,8S)-{(S)-3-[Allyl(t-butoxycarbonyl)amino]-1-(quinolin-7-yl)propyl}3,7-bis(t-butyldimethylsilyloxy)-4,4,6,8-tetra-methyl-5-oxodec-9-enoate* (**17a**). To a solution of alcohol 10 (115 mg, 0.34 mmol) in 3 mL CH_2_Cl_2_ were added sequentially 48 mg (0.39 mmol) of DMAP and 77 mg (0.39 mmol) of EDCI at 0 °C. After stirring for 5 min, a solution of 120 mg (0.24 mmol) of acid **16** [29] in 2 mL CH_2_Cl_2_ was added, the cooling bath was removed and stirring was continued at rt for 5 h, when TLC analysis (hexane/EtOAc 4:1) indicated complete conversion. The mixture was then concentrated in vacuo and the resulting residue was purified by FC (hexane/EtOAc 4:1) to furnish 166 mg (84%) of the desired ester **17a** as a colorless oil; R_f_ = 0.3 (hexane/EtOAc 4:1). ${\left[\alpha \right]}_{D}^{RT}$ = −45.1° (*c* = 1.01, CH_2_Cl_2_). ^1^H-NMR (400 MHz, CDCl_3_): δ = 8.89 (dd, *J* = 4.4 Hz, 1.3 Hz, 1H), 8.11 (d, *J* = 8.4 Hz, 1H), 8.00 (br s, 1H), 7.78 (d, *J* = 8.5 Hz, 1H), 7.50 (d, *J* = 8.2 Hz, 1H), 7.37 (dd, *J* = 8.4 Hz, 4.1 Hz, 1H), 5.86 (m, 2H), 5.69 (m, 1H), 5.06–4.98 (m, 3H), 4.94 (dm, *J* = 17.3 Hz, 1H), 4.31 (dd, *J* = 6.5 Hz, 3.2 Hz, 1H), 3.76 (dd, *J* = 7.1 Hz, 2.0 Hz, 3H), 3.21 (m, 2H), 3.00 (qi, *J* = 7.0 Hz, 1H), 2.50 (dd, *J* = 17.1 Hz, 3.4 Hz, 1H), 2.34 (dd, *J* = 17.1 Hz, 6.1 Hz, 1H), 2.22 (m, 1H), 2.12 (m, 1H), 2.02 (s, 1H), 1.37 (br s, 9H), 1.15 (br s, 3H), 1.00 (s, 3H), 0.98 (d, *J* = 7.0 Hz, 3H), 0.94 (d, *J* = 7.0 Hz, 3H), 0.87 (s, 9H), 0.86 (s, 9H), 0.05 (s, 3H), 0.02 (s, 3H), −0.01 (s, 3H), −0.01 (s, 3H). ^13^C-NMR (100 MHz, CDCl_3_): δ = 217.8, 171.3, 155.2, 150.8, 148.1, 141.4, 139.9, 135.8, 134.1, 128.2, 127.9, 126.9, 125.1, 121.2, 115.3, 116.3, 79.6, 76.2, 74.2, 73.8, 53.3, 50.0, 46.1, 43.6, 43.5, 40.3, 34.9, 28.4 (3 × CH_3_), 26.2 (3 × CH_3_), 26.1 (3 × CH_3_), 23.6, 19.7, 18.8, 18.5, 18.2, 15.2, −3.5, −3.9, −4.2, −4.7. Some of the peaks in the carbon spectrum were hardly visible due to peak broadening. IR (film): ῦ = 2929 (br), 2857, 1738, 1696, 1463, 1365, 1251, 1162, 987, 834, 775, 669 cm^−1^. HRMS (ESIpos): calcd. for C_46_H_76_N_2_O_7_Si_2_Na [M + Na]^+^: 847.5083, found: 847.5088.

*(2S,9S,10S,11R,14S,E)-t-Butyl-10,14-bis(t-butyldimethyl-silyloxy)-9,11,13,13-tetramethyl-12,16-dioxo-2-(quinolin-7-yl)-1-oxa-5-azacyclohexadec-7-ene-5-carboxylate* (**17aA**). To a solution of diene **17a** 63 mg (0.076 mmol) in 148 mL toluene was added Grubbs 2nd generation catalyst (10 mg, 0.012 mmol, 15 mol %) in 2 mL of toluene at reflux temperature. The reddish-yellow solution was stirred at reflux temperature for 30 min, when complete conversion had occurred according to MS analysis; the mixture was then cooled down with an ice bath. The solvent was removed in vacuo and the residue was purified by FC (hexane/EtOAc 10:1 → 4:1). Because the resulting product still had a brownish color, a second column chromatography was performed leading to 50 mg (82%) of the desired olefin **17aA** as a greyish foam; R_f_ = 0.2 (hexane/EtOAc 4:1); R_f_ = 0.5 (hexane/EtOAc 2:1). ${\left[\alpha \right]}_{D}^{RT}$ = −11.2° (*c* = 1.00, CH_2_Cl_2_). ^1^H-NMR (400 MHz, CDCl_3_): δ = 8.90 (dd, *J* = 4.1 Hz, 1.0 Hz, 1H), 8.11 (d, *J* = 8.2 Hz, 1H), 8.08 (br s, 1H), 7.79 (d, *J* = 8.4 Hz, 1H), 7.55 (dd, *J* = 8.4 Hz, 1.6 Hz,1H), 7.37 (dd, *J* = 8.4 Hz, 4.2 Hz, 1H), 6.10 (d, *J* = 9.7 Hz, 1H), 5.37 (dd, *J* = 15.4 Hz, 6.3 Hz, 1H), 5.26 (dm, *J* = 15.6 Hz, 1H), 4.39 (m, 2H), 4.01 (dd, *J* = 8.7 Hz, 2.5 Hz, 1H), 3.64 (m, 1H), 3.25 (m, 2H), 2.99 (m, 1H), 2.60 (dd, *J* = 16.6 Hz, 6.3 Hz, 1H), 2.48 (m, 1H), 2.44–2.38 (m, 1H), 2.30 (m, 1H), 2.20 (m, 1H), 1.40 (s, 9H), 1.24 (s, 3H), 1.15 (s, 3H), 1.13 (d, *J* = 6.8 Hz, 3H), 1.04 (d, *J* = 6.9 Hz, 3H), 0.88 (s, 9H), 0.83 (s, 9H), 0.12 (s, 3H), 0.06 (s, 6H), −0.03 (s, 3H). ^13^C-NMR (100 MHz, CDCl_3_): δ = 216.5, 170.4, 155.6, 150.8, 148.2, 141.5, 135.7, 134.6, 128.2, 127.9, 127.0, 125.2, 124.5, 121.3, 79.6, 77.1, 73.4, 73.0, 54.3, 48.2, 43.8, 43.6, 42.8, 42.5, 34.5, 28.4 (3 × CH_3_), 25.9 (6 × CH_3_), 23.8, 19.0, 18.3, 18.2, 18.1, 12.8, −3.7, −4.3, −4.3, −4.9. Some of the peaks in the carbon spectrum were hardly visible, due to peak broadening. IR (film): ῦ = 2929 (br), 2857, 1739, 1472, 1365, 1252, 1160, 1082, 988, 836, 775, 670 cm^−1^. HRMS (ESIpos): calcd. for C_44_H_72_N_2_O_7_Si_2_Na [M + Na]^+^: 819.4770, found: 847.4766.

*(2S,9S,10S,11R,14S,E)-t-Butyl-10,14-dihydroxy-9,11,13,13-tetramethyl-12,16-dioxo-2-(quinolin-7-yl)-1-oxa-5-azacyclohexadec-7-ene-5-carboxylate* (**5a**). To a solution of bis-TBS-ether **17aA** (116 mg, 0.15 mmol) in 6 mL THF in a 50 mL plastic tube were added 1.3 mL of pyridine and 2 mL of HF∙pyridine (HF∙pyridine dropwise) at 0 °C. After 15 min, the cooling bath was removed and the mixture was stirred at rt for 2 h. As significant amounts of starting material were still detectable at this point by MS analysis, another 1.3 mL of HF∙pyridine were added; 2 h later the conversion to the mono-TBS-protected product was nearly complete. Another 0.8 mL of HF∙pyridine were added and stirring was continued. Subsequent to this, samples of the reaction mixture were analyzed by MS every 30 min. After 7 h only small amounts of the bis-TBS-protected product were still detectable. To avoid BOC-cleavage, the reaction was quenched at this point. Thus, the reaction mixture was added dropwise to 35 mL of a cooled saturated, aqueous NaHCO_3_ solution (strong gas formation). The solution was then extracted with EtOAc and the combined organic layers were washed with brine, dried over MgSO_4_ and concentrated in vacuo. The residue was purified by FC (CH_2_Cl_2_/MeOH 200:1 → 20:1) followed by preparative RP-HPLC on a Waters Symmetry^®^ C18 5 μm (Waters, Milford, MA, USA), 19 mm × 100 mm column, employing an CH_3_CN/H_2_O gradient (CH_3_CN/H_2_O 40:60 → 90:10) at a flow rate of 25 mL/min to furnish 57 mg (67%) of the macrolactone **5a** as a white solid; R_f_ = 0.3 (EtOAc). ${\left[\alpha \right]}_{D}^{\mathrm{20}}$ = −107.4° (*c* = 1.00, CH_2_Cl_2_). ^1^H-NMR (500 MHz, 318 K, DMSO-*d*_6_): δ = 8.89 (dd, *J* = 4.2 Hz, 1.7 Hz, 1H), 8.33 (dm, *J* = 8.4 Hz, 1H), 8.04 (br s, 1H), 7.95 (d, *J* = 8.5 Hz, 1H), 7.62 (dd, *J* = 8.4 Hz, 1.5 Hz, 1H), 7.50 (dd, *J* = 8.2 Hz, 4.2 Hz, 1H), 5.93 (m, 2H), 5.37 (m, 1H), 5.17 (d, *J* = 6.6 Hz, 1H, OH), 4.53 (m, 2H), 4.01 (dd, *J* = 14.9 Hz, 5.3 Hz, 1H), 3.68 (dd, *J* = 15.2 Hz, 7.6 Hz, 1H), 3.56 (m, 1H), 3.28 (m, 2H), 3.12 (m, 1H), 2.45–2.38 (m, 2H), 2.20 (m, 1H), 2.09 (m, 1H), 2.02 (m, 1H), 1.30 (s, 9H), 1.19 (s, 3H), 1.12 (d, *J* = 6.6 Hz, 3H), 1.04 (d, *J* = 6.8 Hz, 3H), 0.93 (s, 3H). ^13^C-NMR (125 MHz, 318 K, DMSO-*d*_6_): δ = 216.6, 169.7, 154.3, 150.5, 147.4, 141.6, 135.6, 135.5, 128.0, 127.0, 125.5, 125.1, 124.2, 121.2, 78.5, 74.4, 73.3, 69.2, 53.4, 48.3, 43.8, 41.0, 40.4, 38.5, 34.3, 27.8 (3 × CH_3_), 19.9, 18.9, 17.3, 14.8. Some of the carbon signals were hardly visible, due to peak broadening. IR (film): ῦ = 3472 (br), 2929 (br), 1740, 1687, 1415, 1365, 1251, 1173, 1042, 982 cm^−1^. HRMS (ESIpos): calcd. for C_32_H_44_N_2_O_7_Na [M + Na]^+^: 591.3041, found: 591.3042.

*(2S,9S,10S,11R,14S)-t-Butyl-10,14-dihydroxy-9,11,13,13-tetramethyl-12,16-dioxo-2-(quinolin-7-yl)-1-oxa-5-azacyclohexadec-ane-5-carboxylate* (**3a**). To a yellow suspension of macrolactone **5a** (9 mg, 0.016 mmol) and dipotassium azadicarboxylate (PADA) (500 mg, 2.58 mmol) in 5 mL CH_2_Cl_2_ was added a solution of AcOH (0.3 mL, 5.25 mmol) in 3 mL CH_2_Cl_2_ dropwise via syringe pump at rt over a period of 2.5 h. After stirring for 18 h, the white suspension was filtered through a plug of Celite™, the residue was washed with CH_2_Cl_2_ and the combined filtrates were concentrated in vacuo. The resulting crude mixture was again dissolved in 5 mL of CH_2_Cl_2_ together with 500 mg (2.58 mmol) PADA and another 0.3 mL (5.25 mmol) AcOH were added as a solution in 3 mL CH_2_Cl_2_ dropwise via syringe pump. After 18 h, the reaction mixture was again filtered. The reaction/work-up-sequence was repeated until a reasonable conversion to the reduced product could be observed by HPLC. Altogether 3.5 g (18 mmol) of PADA and 2.1 mL (36.7 mmol) of AcOH were used, the overall reaction time was 7 days. After the final work up, the resulting crude mixture was purified by preparative RP-HPLC on a Waters Symmetry^®^ C18 5 μm, 19 mm × 100 mm column, employing an CH_3_CN/H_2_O gradient (CH_3_CN/H_2_O 40:60 → 90:10) at a flow rate of 25 mL/min, to yield 6 mg (66%) of the desired azathilone **3a** as a white solid; R_f_ = 0.2 (CH_2_Cl_2_/MeOH 50:1). ${\left[\alpha \right]}_{D}^{RT}$ = −8.7° (*c* = 1.00, CH_2_Cl_2_). ^1^H-NMR (500 MHz, 318 K, DMSO-*d*_6_): δ = 8.89 (dd, *J* = 4.1 Hz, 1.6 Hz, 1H), 8.33 (dm, *J* = 8.3 Hz, 1H), 8.05 (br s, 1H), 7.95 (d, *J* = 8.4 Hz, 1H), 7.63 (dd, *J* = 8.4 Hz, 1.4 Hz, 1H), 7.50 (dd, *J* = 8.3 Hz, 4.2 Hz, 1H), 5.93 (m, 1H), 5.26 (d, *J* = 7.6 Hz, 1H, OH), 4.26 (d, *J* = 6.4 Hz, 1H, OH), 4.19 (m, 1H), 3.43 (m, 1H), 3.37 (m, 1H), 3.23 (m, 2H), 3.10 (m, 1H), 2.96 (m, 1H), 2.42 (dd, *J* = 14.4 Hz, 10.6 Hz, 1H), 2.11 (m, 2H), 1.55 (m, 1H), 1.43 (m, 1H), 1.33 (s, 9H), 1.32 (s, 3H), 1.30–1.16 (m, 4H), 1.02 (d, *J* = 6.7 Hz, 3H), 0.93 (s, 3H), 0.90 (d, *J* = 6.8 Hz, 3H). ^13^C-NMR (125 MHz, 318 K, DMSO-*d*_6_): δ = 217.8, 170.5, 154.3, 150.6, 147.5, 141.7, 135.5, 128.0, 127.0, 125.1, 124.3, 121.2, 78.1, 74.0, 73.1, 71.3, 52.7, 47.0, 44.2, 42.2, 38.6, 36.0, 35.5, 27.9 (3 × CH_3_), 27.0, 24.7, 20.5, 20.0, 17.2, 14.1. Some of the carbon signals were hardly visible, due to peak broadening. IR (film): ῦ = 3442 (br), 2922 (br), 1735, 1687, 1457, 1418, 1366, 1291, 1253, 1160, 1072, 1047, 976, 837 cm^−1^. HRMS (ESIpos): calcd. for C_32_H_46_N_2_O_7_Na [M + Na]^+^: 593.3197, found: 593.3201.

*(3S,6R,7S,8S)-((S)-3-(Allyl(ethoxycarbonyl)amino)-1-(quinolin-7-yl)propyl) 3,7-bis(t-butyl-dimethylsilyloxy)-4,4,6,8-tetra-methyl-5-oxodec-9-enoate* (**17b**). To a solution of alcohol **11** (19 mg, 0.06 mmol) in 2 mL CH_2_Cl_2_ were added sequentially 8.2 mg (0.067 mmol) of DMAP and 13.1 mg (0.067 mmol) of EDCI at 0 °C. After stirring for 5 min, a solution of 21 mg (0.042 mmol) of acid **16** [29] in 2 mL CH_2_Cl_2_ was added, the cooling bath was removed and stirring was continued at rt for 5 h, when TLC analysis (hexane/EtOAc 4:1) indicated complete conversion. The mixture was then concentrated in vacuo and the resulting residue was purified by FC (hexane/EtOAc 4:1) to furnish 28 mg (84%) of the desired ester **17b** as a colorless oil; R_f_ = 0.1 (hexane/EtOAc 4:1). ${\left[\alpha \right]}_{D}^{RT}$ = −37.9° (*c* = 1.00, CH_2_Cl_2_). ^1^H-NMR (400 MHz, CDCl_3_): δ = 8.89 (dd, *J* = 4.3 Hz, 1.0 Hz, 1H), 8.11 (d, *J* = 8.1 Hz, 1H), 8.01 (br s, 1H), 7.78 (d, *J* = 8.3 Hz, 1H), 7.51 (d, *J* = 7.7 Hz, 1H), 7.37 (dd, *J* = 8.4 Hz, 4.1 Hz, 1H), 5.90–5.82 (m, 2H), 5.70 (m, 1H), 5.07 (m, 1H), 5.04 (dm, *J* = 11.5 Hz, 1H), 4.99 (dd, *J* = 10.6 Hz, 1.2 Hz, 1H), 4.94 (dm, *J* = 17.5 Hz, 1H), 4.31 (dd, *J* = 6.4 Hz, 3.4 Hz, 1H), 4.06 (q, *J* = 7.1 Hz, 2H), 3.80 (m, 2H), 3.75 (dd, *J* = 7.2 Hz, 2.0 Hz, 1H), 3.26 (m, 2H), 3.00 (qi, *J* = 7.1 Hz, 1H), 2.50 (dd, *J* = 16.9 Hz, 3.5 Hz, 1H), 2.35 (dd, *J* = 16.7 Hz, 6.4 Hz, 1H), 2.23 (m, 1H), 2.14 (m, 1H), 2.02 (m, 1H), 1.16 (br t, *J* = 7.1 Hz, 3H), 1.15 (s, 3H), 1.00 (s, 3H), 0.98 (d, *J* = 7.2 Hz, 3H), 0.94 (d, *J* = 7.0 Hz, 3H), 0.87 (s, 9H), 0.85 (s, 9H), 0.05 (s, 3H), 0.00 (s, 3H), −0.01 (s, 3H), −0.01 (s, 3H). ^13^C-NMR (100 MHz, CDCl_3_): δ = 217.8, 171.3, 156.1, 150.7, 148.1, 141.5, 139.9, 135.7, 133.7, 128.2, 127.9, 126.9, 125.0, 121.2, 116.8, 115.3, 76.2, 74.0, 73.7, 61.3, 53.3, 49.9, 46.1, 43.4, 43.0, 40.3, 34.8, 26.2 (3 × CH_3_), 26.0 (3 × CH_3_), 23.6, 19.7, 18.8, 18.5, 18.2, 15.2, 14.6, −3.6, −3.9 −4.2, −4.7. Some of the signals in the carbon spectrum were hardly visible, due to peak broadening. IR (film): ῦ = 2955, 2931, 2855, 1739, 1699, 1472, 1416, 1384, 1252, 1171, 988, 836, 774 cm^−1^. HRMS (ESIpos): calcd. for C_44_H_72_N_2_O_7_Si_2_Na [M + Na]^+^: 819.4770, found: 819.4772.

*(2S,9S,10S,11R,14S,E)-Ethyl 10,14-bis(t-butyldimethyl-silyloxy)-9,11,13,13-tetramethyl-12,16-dioxo-2-(quinolin-7-yl)-1-oxa-5-azacyclohexadec-7-ene-5-carboxylate* (**17bA**). To a solution of diene **17b** (25 mg, 0.031 mmol) in 63 mL toluene was added Grubbs 2nd generation catalyst (4.0 mg, 0.0047 mmol, 15 mol %) in 2 mL of toluene at reflux temperature. The reddish-yellow solution was stirred at reflux temperature for 30 min, when complete conversion had occurred according to MS analysis; the mixture was then cooled down with an ice bath. The solvent was removed in vacuo and the residue was purified by FC (hexane/EtOAc 4:1) to furnish 23 mg (96%) of the desired olefin **17bA** as a brown oil. ${\left[\alpha \right]}_{D}^{RT}$ = −8.6° (*c* = 1.00, CH_2_Cl_2_). ^1^H-NMR (400 MHz, CDCl_3_): δ = 8.91 (dm, *J* = 3.9 Hz, 1H), 8.12 (d, *J* = 8.4 Hz, 1H), 8.10 (s, 1H), 7.79 (d, *J* = 8.1 Hz, 1H), 7.56 (dd, *J* = 8.5 Hz, 1.7 Hz, 1H), 7.38 (dd, *J* = 8.5 Hz, 4.0 Hz, 1H), 6.11 (d, *J* = 10.5 Hz, 1H), 5.38 (dd, *J* = 15.7 Hz, 6.3 Hz, 1H), 5.30–5.23 (m, 1H), 4.47–4.38 (m, 2H), 4.10 (m, 2H), 4.00 (dd, *J* = 8.7 Hz, 2.9 Hz, 1H), 3.71 (m, 1H), 3.29 (m, 2H), 2.99 (m, 1H), 2.60 (dd, *J* = 16.8 Hz, 6.2 Hz, 1H), 2.48 (m, 1H), 2.43–2.38 (m, 1H), 2.32–2.16 (m, 2H), 1.24 (s, 3H), 1.20 (br t, *J* = 7.1 Hz, 3H), 1.15 (s, 3H), 1.13 (d, *J* = 6.8 Hz, 3H), 1.04 (d, *J* = 6.9 Hz, 3H), 0.88 (s, 9H), 0.83 (s, 9H), 0.12 (s, 3H), 0.07 (s, 6H), −0.04 (s, 3H). ^13^C-NMR (100 MHz, CDCl_3_): δ = 216.3, 170.4, 150.8, 148.2, 144.3, 141.5, 135.7, 135.0, 128.1, 127.9, 127.2, 125.5, 124.2, 121.3, 77.2, 73.3, 73.0, 61.3, 54.3, 48.7, 43.8, 43.3, 42.9, 42.5, 34.3, 25.9 (6xCH_3_), 23.9, 19.1, 18.4, 18.2, 18.1, 14.7, 13.1, −3.7, −4.2 (2 × CH_3_), −4.9. Some of the signals in the carbon spectrum were hardly visible, due to peak broadening (especially the quaternary C-atoms). IR (film): ῦ = 2931, 2855, 2354, 1739, 1695, 1472, 1424, 1385, 1250, 1216, 1084, 988, 836, 774 cm^−1^. HRMS (ESIpos): calcd. for C_42_H_68_N_2_O_7_Si_2_Na [M + Na]^+^: 791.4457, found: 791.4464.

*(2S,9S,10S,11R,14S,E)-Ethyl 10,14-dihydroxy-9,11,13,13-tetramethyl-12,16-dioxo-2-(quinolin-7-yl)-1-oxa-5-azacyclohexadec-7-ene-5-carboxylate* (**5b**). To a solution of bis-TBS-ether **17bA** (22 mg, 0.029 mmol) in 1.5 mL THF in a 12 mL plastic tube were added 0.25 mL of pyridine and 0.4 mL of HF∙pyridine (HF∙pyridine dropwise) at 0 °C. After 15 min, the cooling bath was removed and the mixture was stirred at rt for 2 h. As significant amounts of starting material were still detectable at this point by MS analysis, another 0.3 mL of HF∙pyridine were added; 4 h later the conversion was still incomplete and another 0.1 mL of HF∙pyridine were added. 3 h later MS analysis indicated the presence of product and only traces of the mono-TBS-deprotected species. The reaction mixture was then added dropwise to a cooled saturated, aqueous NaHCO_3_ solution (strong gas formation). The solution was then extracted with EtOAc and the combined organic layers were washed with brine, dried over MgSO_4_ and concentrated in vacuo. The residue was purified by FC (CH_2_Cl_2_/MeOH 100:1 → 20:1) followed by preparative RP-HPLC on a Waters Symmetry^®^ C18 5 μm, 19 mm × 100 mm column, employing an CH_3_CN/H_2_O gradient (CH_3_CN/H_2_O 20:80 → 95:5) at a flow rate of 25 mL/min to furnish 8 mg (51%) of the macrolactone **5b** as a white solid; R_f_ = 0.6 (CH_2_Cl_2_/MeOH 10:1). ${\left[\alpha \right]}_{D}^{RT}$ = −132.7° (*c* = 1.00, CH_2_Cl_2_). ^1^H-NMR (500 MHz, 318 K, DMSO-*d*_6_): δ = 8.90 (dd, *J* = 4.2 Hz, 1.7 Hz, 1H), 8.33 (dd, *J* = 8.4 Hz, 1.4 Hz, 1H), 8.05 (d, *J* = 0.9 Hz, 1H), 7.96 (d, *J* = 8.4 Hz, 1H), 7.63 (dd, *J* = 8.4 Hz, 1.7 Hz, 1H), 7.51 (dd, *J* = 8.3 Hz, 4.2 Hz, 1H), 5.95 (m, 2H), 5.38 (m, 1H), 5.15 (d, *J* = 6.6 Hz, 1H, OH), 4.56 (d, *J* = 6.4 Hz, 1H, OH), 4.52 (m, 1H), 4.08 (dd, *J* = 14.8 Hz, 5.2 Hz, 1H), 3.98 (q, *J* = 7.0 Hz, 2H), 3.71 (dd, *J* = 15.1 Hz, 7.8 Hz, 1H), 3.56 (dt, *J* = 7.0 Hz, 3.2 Hz, 1H), 3.37 (m, 1H), 3.27 (qi, *J* = 7.1 Hz, 1H), 2.47–2.39 (m, 2H), 2.22 (m, 1H), 2.10 (m, 1H), 2.04 (m, 2H), 1.18 (s, 3H), 1.12 (d, *J* = 6.7 Hz, 3H), 1.11 (t, *J* = 7.1 Hz, 3H), 1.04 (d, *J* = 6.8 Hz, 3H), 0.93 (s, 3H). ^13^C-NMR (125 MHz, 318 K, DMSO-*d*_6_): δ = 216.6, 169.8, 155.1, 150.6, 147.4, 141.6, 135.8, 135.5, 128.0, 127.0, 125.3, 125.2, 124.3, 121.2, 74.4, 73.3, 69.2, 60.4, 53.4, 48.4, 43.9, 41.1, 39.5, 38.5, 34.1, 19.9, 19.0, 17.4, 14.9, 14.3. Some of the signals in the carbon spectrum were hardly visible, due to peak broadening (especially the quaternary C-atoms). IR (film): ῦ = 3446 (br), 2976, 2927, 1739, 1689, 1468, 1421, 1383, 1288, 1251, 1221, 1181, 1046, 983, 890, 838, 771 cm^−1^. HRMS (ESIpos): calcd. for C_30_H_40_N_2_O_7_Na [M + Na]^+^: 563.2728, found: 563.2728.

*(2S,9S,10S,11R,14S)-Ethyl 10,14-dihydroxy-9,11,13,13-tetramethyl-12,16-dioxo-2-(quinolin-7-yl)-1-oxa-5-azacyclohexadec-ane-5-carboxylate* (**3b**). To a mixture of macrolactone **5b** (4 mg, 0.0074 mmol) and dipotassium azadicarboxylate (PADA) (1.0 g, 5.15 mmol) in 5 mL CH_2_Cl_2_ was added a solution of AcOH (0.6 mL, 5.25 mmol) in 2.5 mL CH_2_Cl_2_ dropwise via syringe pump at rt over a period of 2.5 h. After stirring for 1 d, the mixture was filtered through a plug of Celite™, the filtrate was concentrated in vacuo and the residue was again treated with 1 g of PADA and 0.6 mL of AcOH as described above. After 2 days, analytical RP-HPLC showed the presence of 60% starting material and 40% product. The mixture was worked up as described above and the crude product mixture was again treated with 1 g of PADA and 0.6 mL of AcOH as described above and the mixture was stirred at room temperature for another 2.5 days. After this time only 40% of starting material were present in the reaction mixture. A last work-up/treatment with 1 g of PADA and 0.6 mL of AcOH cycle and stirring for another day resulted in a starting material/product ratio of ~25:75. The mixture was filtered, washed with H_2_O, extracted with CH_2_Cl_2_, dried over MgSO_4_ and concentrated. The resulting crude product mixture was purified by semi-preparative HPLC (Waters Symmetry^®^ C18 5 μm, 7.8 mm × 100 mm column; gradient from CH_3_CN/H_2_O 20:80 to 90:10; 3 mL/min flow rate) to provide 1 mg (25%) of re-isolated starting material **5b** and 2.6 mg (65%) of the desired macrolactone **3b** both as white solids. R_f_ = 0.2 (CH_2_Cl_2_/MeOH 50:1); R_f_ = 0.6 (CH_2_Cl_2_/MeOH 10:1). ${\left[\alpha \right]}_{D}^{RT}$ = −9.6° (*c* = 1.00, CH_2_Cl_2_). ^1^H NMR (500 MHz, 318 K, DMSO-*d*_6_): δ = 8.89 (dd, *J* = 4.2 Hz, 1.7 Hz, 1H), 8.33 (dm, *J* = 8.5 Hz, 1H), 8.06 (br s, 1H), 7.95 (d, *J* = 8.5 Hz, 1H), 7.64 (dd, *J* = 8.6 Hz, 1.8 Hz, 1H), 7.50 (dd, *J* = 8.2 Hz, 4.1 Hz, 1H), 5.93 (dd, *J* = 7.1 Hz, 4.2 Hz, 1H), 5.25 (d, *J* = 7.4 Hz, 1H, OH), 4.27 (d, *J* = 6.3 Hz, 1H, OH), 4.19 (m, 1H), 3.97 (q, *J* = 7.0 Hz, 2H), 3.58 (m, 1H), 3.51 (m, 1H), 3.43 (m, 1H), 3.22–3.15 (m, 3H), 3.01 (m, 1H), 2.40 (dd, *J* = 14.7 Hz, 10.7 Hz, 1H), 2.12 (m, 2H), 1.57 (m, 1H), 1.45 (m, 1H), 1.39–1.30 (m, 1H), 1.32 (s, 3H), 1.27–1.22 (m, 2H), 1.12 (t, *J* = 7.0 Hz, 3H), 1.02 (d, *J* = 6.7 Hz, 3H), 0.93 (s, 3H), 0.90 (d, *J* = 6.9 Hz, 3H). ^13^C-NMR (125 MHz, 318 K, DMSO-*d*_6_): δ = 217.7, 170.5, 155.1, 150.6, 147.4, 141.7, 135.5, 128.0, 127.0, 125.2, 124.4, 121.2, 73.9, 73.0, 71.5, 60.2, 52.7, 46.9, 44.2, 42.1, 38.6, 36.0, 35.1, 26.9, 24.6, 20.5, 20.0, 17.2, 14.4, 14.1. Some of the signals in the carbon spectrum were hardly visible, due to peak broadening. Several of the carbon signals were also duplicated, which we ascribe to the existence of rotamers. In these cases only the signal for the major rotamer is listed. IR (film): ῦ = 3408 (br), 2922 (br), 2851, 1735, 1687, 1457, 1428, 1386, 1290, 1249, 1214, 1148, 1048, 977, 837 cm^−1^. HRMS (ESIpos): calcd. for C_30_H_43_N_2_O_7_ [M + H]^+^: 543.3065, found: 543.3064.

*(3S,6R,7S,8S)-((S)-3-(Allyl(ethoxycarbonyl)amino)-1-(quinolin-6-yl)propyl) 3,7-bis(t-butyl-dimethylsilyloxy)-4,4,6,8-tetra-methyl-5-oxodec-9-enoate* (**18**). To a solution of alcohol **15** (48 mg, 0.14 mmol) in 3 mL CH_2_Cl_2_ were added sequentially 20 mg (0.16 mmol) of DMAP and 32 mg (0.16 mmol) of EDCI at 0 °C. After stirring for 5 min, a solution of 50 mg (0.1 mmol) of acid **16** [29] in 2 mL CH_2_Cl_2_ was added, the cooling bath was removed and stirring was continued at rt for 5 h, when TLC analysis (hexane/EtOAc 4:1) indicated complete conversion. The mixture was then concentrated in vacuo and the resulting residue was purified by FC (hexane/EtOAc 4:1) to furnish 74 mg (90%) of the desired ester **18** as a colorless oil; R_f_ = 0.4 (hexane/EtOAc 4:1). ${\left[\alpha \right]}_{D}^{RT}$ = −42.0° (*c* = 1.00, CH_2_Cl_2_). ^1^H-NMR (400 MHz, CDCl_3_): δ = 8.88 (dm, *J* = 4.4 Hz, 1H), 8.12 (dm, *J* = 8.8 Hz, 1H), 8.07 (d, *J* = 8.8 Hz, 1H), 7.75 (br s, 1H), 7.66 (dd, *J* = 8.8 Hz, 1.7 Hz, 1H), 7.38 (dd, *J* = 8.1 Hz, 4.3 Hz, 1H), 5.85 (m, 2H), 5.70 (m, 1H), 5.07–4.98 (m, 3H), 4.94 (dm, *J* = 17.6 Hz, 1H), 4.28 (dd, *J* = 6.0 Hz, 3.7 Hz, 1H), 3.75 (dd, *J* = 7.0 Hz, 2.0 Hz, 3H), 3.21 (m, 2H), 3.02 (qi, *J* = 7.0 Hz, 1H), 2.50 (dd, *J* = 17.1 Hz, 3.4 Hz, 1H), 2.32 (dd, *J* = 17.1 Hz, 6.3 Hz, 1H), 2.21 (m, 1H), 2.11 (m, 1H), 2.04 (m, 1H), 1.38 (br s, 9H), 1.15 (s, 3H), 0.99 (d, *J* = 7.0 Hz, 3H), 0.98 (s, 3H), 0.94 (d, *J* = 7.0 Hz, 3H), 0.87 (s, 9H), 0.85 (s, 9H), 0.05 (s, 3H), 0.00 (s, 3H), −0.01 (s, 3H), −0.01 (s, 3H). ^13^C-NMR (100 MHz, CDCl_3_): δ = 217.9, 171.3, 155.2, 150.6, 148.0, 139.8, 138.1, 136.2, 134.1, 130.0, 128.0, 127.6, 125.7, 121.4, 116.6, 115.3, 79.7, 76.2, 74.1, 73.9, 53.3, 49.9, 46.1, 43.5, 43.5, 40.4, 34.7, 28.4 (3 × CH_3_), 26.2 (3 × CH_3_), 26.0 (3 × CH_3_), 23.6, 19.8, 18.7, 18.5, 18.2, 15.1, −3.6, −3.9, −4.3, −4.7. Some of the signals in the carbon spectrum were hardly visible, due to peak broadening. IR (film): ῦ = 2929 (br), 2857, 1739, 1697, 1463, 1365, 1251, 1169, 988, 836, 776, 669 cm^−1^. HRMS (ESIpos): calcd. for C_46_H_76_N_2_O_7_Si_2_Na [M+Na]^+^: 847.5083, found: 847.5080.

*(2S,9S,10S,11R,14S,E)-Ethyl 10,14-bis(t-butyldimethyl-silyloxy)-9,11,13,13-tetramethyl-12,16-dioxo-2-(quinolin-6-yl)-1-oxa-5-azacyclohexadec-7-ene-5-carboxylate* (**18A**). To a solution of diene **18** (73 mg, 0.088 mmol) in 148 mL toluene was added Grubbs 2nd generation catalyst (10 mg, 0.012 mmol, 15 mol %) in 2 mL of toluene at reflux temperature. The reddish-yellow solution was stirred at reflux temperature for 30 min, when complete conversion had occurred according to MS analysis; the mixture was then cooled down with an ice bath. The solvent was removed in vacuo and the residue was purified by FC (hexane/EtOAc 10:1 → 4:1) to furnish 58 mg (82%) of the desired olefin **18A** as a light brown foam; R_f_ = 0.3 (hexane/EtOAc 4:1); R_f_ = 0.6 (hexane/ EtOAc 2:1). ${\left[\alpha \right]}_{D}^{RT}$ = −7.2° (*c* = 1.00, CH_2_Cl_2_). ^1^H-NMR (400 MHz, CDCl_3_): δ = 8.89 (d, *J* = 3.0 Hz, 1H), 8.12 (d, *J* = 7.9 Hz, 1H), 8.07 (d, *J* = 8.9 Hz, 1H), 7.81 (d, *J* = 1.8 Hz, 1H), 7.71 (dd, *J* = 8.9 Hz, 1.8 Hz, 1H), 7.39 (dd, *J* = 8.3 Hz, 4.1 Hz, 1H), 6.08 (d, *J* = 10.7 Hz, 1H), 5.34 (dd, *J* = 15.7 Hz, 6.3 Hz, 1H), 5.29–5.22 (m, 1H), 4.47–4.24 (m, 2H), 4.01 (dd, *J* = 8.7 Hz, 2.5 Hz, 1H), 3.64 (m, 1H), 3.24 (dd, *J* = 15.9 Hz, 1.3 Hz, 2H), 2.99 (m, 1H), 2.58 (dd, *J* = 16.8 Hz, 6.1 Hz, 1H), 2.48 (m, 1H), 2.37 (dd, *J* = 16.9 Hz, 2.5 Hz, 1H), 2.30–2.13 (m, 2H), 1.40 (s, 9H), 1.25 (s, 3H), 1.15 (s, 3H), 1.13 (d, *J* = 6.8 Hz, 3H), 1.04 (d, *J* = 7.0 Hz, 3H), 0.88 (s, 9H), 0.84 (s, 9H), 0.13 (s, 3H), 0.07 (s, 6H), −0.04 (s, 3H); ^13^C-NMR (100 MHz, CDCl_3_): δ = 216.4, 170.4, 155.5, 150.7, 148.0, 138.4, 136.1, 134.6, 129.9, 128.0, 127.9, 125.7, 124.5, 121.4, 79.6, 77.2, 73.3, 72.9, 54.3, 48.3, 43.7, 43.5, 43.0, 42.6, 34.5, 28.4 (3 × CH_3_), 25.9 (6 × CH_3_), 23.9, 18.9, 18.4, 18.2, 18.1, 12.7, −3.7, −4.3, −4.3, −4.9. Some of the signals in the carbon spectrum were hardly visible, due to peak broadening. IR (film): ῦ = 2928 (br), 2857, 1738, 1693, 1472, 1365, 1251, 1162, 1082, 988, 836, 775, 666 cm^−1^. HRMS (ESIpos): calcd. for C_44_H_72_N_2_O_7_Si_2_Na [M + Na]^+^: 819.4770, found: 847.4773.

*(2S,9S,10S,11R,14S,E)-Ethyl 10,14-dihydroxy-9,11,13,13-tetramethyl-12,16-dioxo-2-(quinolin-6-yl)-1-oxa-5-azacyclohexadec-7-ene-5-carboxylate* (**6**). To a solution of bis-TBS-ether **18A** (56 mg, 0.07 mmol mmol) in 2.5 mL THF in a 12 mL plastic tube were added 0.5 mL of pyridine and 1.5 mL of HF∙pyridine (HF∙pyridine dropwise) at 0 °C. After 20 min, the cooling bath was removed and the mixture was stirred at rt for 2 h. As significant amounts of starting material and mono-protected intermediate were still detectable at this point by MS analysis, another 0.3 mL of HF∙pyridine were added; 4 h later the reaction mixture was added dropwise to a cooled saturated, aqueous NaHCO_3_ solution (strong gas formation). The solution was then extracted with EtOAc and the combined organic layers were washed with brine, dried over MgSO_4_ and concentrated in vacuo. The residue was purified by FC (CH_2_Cl_2_/MeOH 200:1 → 20:1) followed by preparative RP-HPLC (twice) on a Waters Symmetry^®^ C18 5 μm, 19 mm × 100 mm column, employing an CH_3_CN/H_2_O gradient (CH_3_CN/H_2_O 40:60 → 90:10) at a flow rate of 25 mL/min to furnish 22 mg (55%) of the macrolactone **6** as a white solid; R_f_ = 0.4 (EtOAc). ${\left[\alpha \right]}_{D}^{RT}$ = −111.3° (*c* = 1.00, CH_2_Cl_2_). ^1^H-NMR (500 MHz, 318 K, DMSO-*d*_6_): δ = 8.88 (dd, *J* = 4.1 Hz, 1.7 Hz, 1H), 8.31 (dm, *J* = 8.5 Hz, 1H), 8.00 (d, *J* = 8.7 Hz, 1H), 7.97 (d, *J* = 1.6 Hz, 1H), 7.77 (dd, *J* = 8.7 Hz, 1.8 Hz, 1H), 7.52 (dd, *J* = 8.4 Hz, 4.2 Hz, 1H), 5.95 (dd, *J* = 15.6 Hz, 5.2 Hz, 1H), 5.91 (dd, *J* = 7.3 Hz, 3.4 Hz, 1H), 5.36 (m, 1H), 5.19 (d, *J* = 6.5 Hz, 1H, OH), 4.55 (d, *J* = 6.3 Hz, 1H), 4.53–4.50 (m, 1H), 4.00 (dd, *J* = 14.8 Hz, 5.4 Hz, 1H), 3.68 (dd, *J* = 14.7 Hz, 7.6 Hz, 1H), 3.56 (m, 1H), 3.29 (m, 2H), 3.09 (m, 1H), 2.45 (dd, *J* = 15.6 Hz, 4.8 Hz, 1H), 2.38 (dd, *J* = 15.4 Hz, 8.2 Hz, 1H), 2.18 (m, 1H), 2.10 (m, 1H), 2.02 (m, 1H), 1.31 (br s, 9H), 1.22 (s, 3H), 1.11 (d, *J* = 6.7 Hz, 3H), 1.05 (d, *J* = 6.9 Hz, 3H), 0.93 (s, 3H). ^13^C-NMR (125 MHz, 318 K, DMSO-*d*_6_): δ = 216.5, 169.7, 154.2, 150.3, 147.0, 138.4, 136.0, 135.8, 128.8, 127.3, 127.2, 125.3, 124.1, 121.5, 78.5, 74.4, 73.2, 69.3, 53.5, 48.4, 43.7, 41.0, 39.3, 38.4, 34.3, 27.8 (3 × CH_3_), 20.3, 18.7, 17.1, 14.6. Some of the signals in the carbon spectrum were hardly visible, due to peak broadening. IR (film): ῦ = 3383 (br), 2925 (br), 1734, 1687, 1462, 1413, 1366, 1254, 1164, 1010, 985, 836; HRMS (ESIpos): calcd. for C_32_H_44_N_2_O_7_Na [M + Na]^+^: 591.3041, found: 591.3048.

*(2S,9S,10S,11R,14S)-Ethyl 10,14-dihydroxy-9,11,13,13-tetramethyl-12,16-dioxo-2-(quinolin-7-yl)-1-oxa-5-azacyclohexadec-ane-5-carboxylate* (**4**). To a yellow suspension of macrolactone **6** (9 mg, 0.016 mmol) and dipotassium azadicarboxylate (PADA) (307 mg, 1.6 mmol) in 1 mL MeOH and 0.2 mL CH_2_Cl_2_ was added a solution of AcOH (0.18 mL) in 4 mL MeOH dropwise via syringe pump at rt over a period of 4 h. After stirring for 14 h, the mixture was filtered through a plug of Celite™, the filtrate was concentrated in vacuo and the residue was redissolved in 1 mL MeOH and 0.2 mL CH_2_Cl_2_ and another 307 mg of PADA were added. Then a solution of acetic acid (0.18 mL) in 4 mL MeOH was added via syringe pump at rt over a period of 2 h. After this time the white suspension was heated to 50 °C for 5 h. At this point, only a traces of product were detectable by HPLC. The mixture was again filtered through a plug of Celite™ and concentrated in vacuo. The residue was redissolved in 4 mL of MeOH together with 1 g of PADA and a solution of acetic acid (0.6 mL) in 3 mL MeOH was added via syringe pump over a period of 2 h. The mixture was stirred for 14 h and then filtered, the filtrate was concentrated, the residue was dissolved in 4 mL of CH_2_Cl_2_ together with 1 g of PADA and a solution of AcOH (0.6 mL) in 3 mL CH_2_Cl_2_ was added via syringe pump over a 2 h period. The mixture was stirred at room temperature for 2 days and then worked up as above. The residue was dissolved in EtOAc and the solution was washed with water. After drying over MgSO_4_ the organic solution was concentrated in vacuo and the residue was purified twice by preparative RP-HPLC (Waters Symmetry^®^ C18 5 μm, 19 mm × 100 mm column, gradient of CH_3_CN/H_2_O from 20:80 to 90:10 at a 25 mL/min flow rate) and finally be semi-preparative RP-HPLC (Waters Symmetry^®^ C18 5 μm, 7.8 mm × 100 mm column, gradient of CH_3_CN/H_2_O from 20:80 to 90:10 at a 3 mL/min flow rate), to furnish 5 mg (55%) of the desired azathilone **4** and 1.6 mg (18%) of starting material **4** both as white solids; R_f_ = 0.1 (CH_2_Cl_2_/MeOH 50:1). ${\left[\alpha \right]}_{D}^{RT}$ = −17.2° (*c* = 1.00, CH_2_Cl_2_). ^1^H-NMR (500 MHz, 318 K, DMSO-*d*_6_): δ = 8.88 (dd, *J* = 4.2 Hz, 1.7 Hz, 1H), 8.32 (dd, *J* = 8.4 Hz, 1.4 Hz, 1H), 8.00 (d, *J* = 5.9 Hz, 1H), 7.99 (s, 1H), 7.78 (dd, *J* = 8.7 Hz, 1.8 Hz, 1H), 7.53 (dd, *J* = 8.2 Hz, 4.1 Hz, 1H), 5.91 (m, 1H), 5.27 (d, *J* = 7.5 Hz, 1H, OH), 4.27 (d, *J* = 6.7 Hz, 1H, OH), 4.22 (m, 1H), 3.43 (m, 1H), 3.35 (m, 1H), 3.23 (m, 2H), 3.09 (m, 1H), 2.98 (m, 1H), 2.40 (dd, *J* = 14.5 Hz, 10.7 Hz, 1H), 2.11 (m, 2H), 1.56 (m, 1H), 1.46 (m, 1H), 1.33 (s, 12H), 1.32–1.22 (m, 4H), 1.02 (d, *J* = 6.8 Hz, 3H), 0.94 (s, 3H), 0.90 (d, *J* = 6.8 Hz; 3H); ^13^C-NMR (125 MHz, 318 K, DMSO-*d*_6_): δ = 217.6, 170.4, 154.3, 150.3, 147.0, 138.5, 135.8, 128.8, 127.4, 127.3, 124.1, 121.5, 78.1, 73.8, 73.0, 71.3, 52.8, 46.9, 44.1, 42.1, 38.6, 35.9, 35.3, 27.9 (3 × CH_3_), 27.0, 24.4, 20.7, 19.8, 17.2, 13.8. Some of the signals in the carbon spectrum were hardly visible, due to peak broadening. IR (film): ῦ = 3390 (br), 2926 (br), 1733, 1684, 1467, 1420, 1366, 1290, 1253, 1148, 1026, 1010, 977, 837 cm^−1^. HRMS (ESIpos): calcd. for C_32_H_46_N_2_O_7_Na [M + Na]^+^: 593.3197, found: 593.3196.

### 3.3. Antiproliferative Activity

The experiments were essentially carried out as described in ref. [49]: Cells were maintained in a 5% CO_2_ humidified atmosphere at 37 °C in RPMI medium 1640 (Gibco BRL, San Francisco, CA, USA) containing 10% fetal bovine serum, penicillin (100 U/mL) and streptomycin (100 μg/mL) (Gibco BRL). Cells were seeded at 1.5 × 10^3^/well into 96-well microtiter plates and incubated overnight. Compounds were added in serial dilutions on day 1. Subsequently, the plates were incubated for 72 h and then fixed with 3.3% *v*/*v* glutaraldehyde, washed with water and stained with 0.05% methylene blue. After washing, the dye was eluted with 3% *v*/*v* HCl and the optical density (OD) measured at 665 nm with a TECAN GeniosPro (Männesdorf, Switzerland). IC_50_ values were determined with Graphpad Prism 4 using the formula (OD_treated_ − OD_start_)/(OD_control_ − OD_start_) × 100. The IC_50_ is the drug concentration for which the total cell number per well corresponds to 50% of the cell number in untreated control cultures (100%) at the end of the incubation period. Data shown in Table 1 represent the mean of three independent experiments.

### 3.4. Determination of Microtubule Binding Constants

Purified calf-brain tubulin and chemicals were obtained as previously described [50]. Stabilized, moderately crosslinked microtubules were prepared as reported in ref. [51]. Binding constants of azathilones to stabilized microtubules were measured as previously described by Matesanz et al. [38].

### 3.5. Conformational Studies

#### 3.5.1. NMR spectroscopy

NMR spectra were recorded at 298 K on a Bruker AVANCE 500 MHz spectrometer equipped with a triple-channel probe. Solution NMR was performed at 300 μM sample concentration in D_2_O. Samples of **2** bound to tubulin α/β-heterodimers were prepared in 3 mm NMR tubes using a 300 μM concentration of the desired compound and 20 μM of tubulin in D_2_O, 10 mM NaPi, 0.1 mM GTP pH* 7.0. The tubulin sample was prepared by removing sucrose, Mg^2+^, and H_2_O from the storage buffer of a 10 mg sample of frozen tubulin, by chromatography using a Sephadex G-25 medium column (Sigma-Aldrich, 25 cm × 0.9 cm) equilibrated in D_2_O, 10 mM NaPi, 0.1 mM GTP pH* 7.0. Tubulin was centrifuged for 10 min at 50.000 rpm in a TLA120 rotor in an Optima TLX centrifuge to remove aggregates, and its concentration was determined spectrophotometrically by employing an extinction coefficient of 107,000 M^−1^·cm^−1^ in 10 mM phosphate buffer containing 1% SDS. The samples were incubated at 25 °C for 30 min prior to measurement. STD experiments were performed with 0.5, 1, and 2 s saturation times (by concatenation of 50 ms Gaussian pulses separated by 1 ms). TR-NOESY experiments were performed with mixing times of 50, 100, 200, 250 and 300 ms. No purging spin lock period to remove the NMR signals of the background macromolecule was employed, since they were basically not observable due to the large size of the protein. First, line broadening of the ligand protons was monitored after the addition of the protein. Strong negative NOE cross peaks were observed, in contrast to the free state, indicating binding of **2** to the tubulin α/β-heterodimer preparation.

The theoretical STD effects for ligands bound to tubulin α/β-heterodimers were calculated using CORCEMA-ST program [48]. The overall correlation time τ_c_ for the free state was always set to 0.75 ns and the average rotational motion correlation time, τ_c_, for the bound state was set to 60 ns for tubulin α/β-heterodimers. An order parameter S^2^ = 0.85 was employed to account for the fast rotation of the methyl groups, as implemented in CORCEMA-ST.

#### 3.5.2. Conformational Search of Ligands

The calculations were performed using the Macromodel/Batchmin package and the OPLS2005 all-atom force field as implemented in the program Macromodel. Bulk water solvation was simulated using Macromodel’s generalized Born GB/SA continuum solvent model. The conformational searches were carried out using the torsional sampling MCMM search method implemented in the Batchmin program, and 20,000 Monte Carlo step runs were performed. Extended non-bonded cutoff distances (a van der Waals cutoff of 8.0 Å and an electrostatic cutoff of 20.0 Å) were used. PR conjugate gradient (PRCG) minimization (90,000 steps) was used in the conformational search.

#### 3.5.3. Docking and CORCEMA-ST calculations

Protein files were prepared with the Protein Preparation Wizard module implemented in Maestro. Docking calculations were carried out with Autodock Vina [47]. A rigid docking protocol was performed first, using the tubulin-bound conformation of **2**, as it had been derived from TR-NOE experiments. Different conformers of the flexible parts were considered later in refinement process. Docking poses were minimized by using Macromodel with OPLS2005 as the force field and several steps of Polak-Ribière conjugate gradient (PRCG) until the energy gradient become lower than 0.05 kJ/mol/Å. The theoretical STD of the refined docking solutions was calculated by using CORCEMA-ST program [48] to select the binding poses that best fit the experimental data. In order to take into account the flexibility of the molecule several conformations were considered for both the aromatic substituent and the tbutyoxycarbonyl group. 144 conformers were built by the torsional scanning of C15-C16 bond and the *O*-C(CH_3_)_3_ bond in the tbutyoxycarbonyl substituent. In each step the binding pose was optimized to avoid steric clashes. Residues located at 10 Å of the ligand were considered flexible in the refinement process. Two additional shells were considered, 10–13 Å around the ligand with constrained atoms and 13–15 Å around the ligand with frozen atoms.

## Figures and Tables

**Table 1:** Antiproliferative activity of azathilones against human cancer cell lines (IC_50_ [nM]) ^1^.

Compound	A549	MCF-7	SK-OV-3	SKVBL1
**2**	1.9 ± 0.4 ^2^	ND	2.7 ± 0.4 ^3^	879 ± 5 ^3^
**3a**	1.1 ± 0.1	1.4 ± 0.2	1.2 ± 0.3	71 ± 3
**5a**	2986 ± 113	3133 ± 147	ND	ND
**3b**	5.4 ± 0.2	5.9 ± 0.2	4.8 ± 0.7	372 ± 3
**5b**	>5000	>5000	ND	ND
**4**	1023 ± 29	998 ± 43	ND	ND
**6**	>>5000	>>5000	ND	ND
Epothilone A	2.8 ± 0.1	2.1 ± 0.3	1.3 ± 0.2	3.9 ± 0.3

^1^ Cells were incubated with test compounds for 72 h. A549, human small cell lung carcinoma; MCF-7, human breast carcinoma; SK-OV-3, human ovarian carcinoma; SKVBL1, Pgp-overexpressing variant of the SK-OV-3 cell line [37]. Numbers presented are average values from three independent experiments ± standard deviation. For experimental details see the supporting materials. ND, not determined; ^2^ From ref. [16]; ^3^ This work.

**Table 2:** Binding constants of azathilones for stabilized microtubules ^1^.

Compound	K_b_ [10^6^·M^−1^]
**2**	10 ± 2
**3a**	4.2 ± 0.6
**5a**	0.43 ± 0.05
**4**	0.30 ± 0.05
**Epothilone A**	36.3 ^2^

^1^ Association constant K_b_ with glutaraldehyde-stabilized microtubules at 35 °C, as determined as described in ref. [38]. Numbers are average values from three independent experiments ± standard deviation; ^2^ From ref. [39].

## Supplementary Materials

Supplementary materials can be accessed at: http://www.mdpi.com/1420-3049/21/8/1010/s1.
