# Supplementary material for: Synthesis, Biological Profiling and Determination of the Tubulin-Bound Conformation of 12-Aza-Epothilones (Azathilones)
Source: Molecules. 2016 Aug 3;21(8):1010. doi: 10.3390/molecules21081010 (PMC6273374; doi:10.3390/molecules21081010)
Supplement: Supplementary file 1 [file molecules-21-01010-s001.pdf]

Supplementary Materials: Synthesis, Biological Profiling and Determination of the Tubulin-bound Conformation of 12-Aza-Epothilones (Azathilones)

Andrea Jantsch, Lidia Nieto, Jürg Gertsch, Javier Rodríguez-Salarichs, Ruth Matesanz, Jesús Jiménez-Barbero, J. Fernando Díaz, Ángeles Canales and Karl-Heinz Altmann

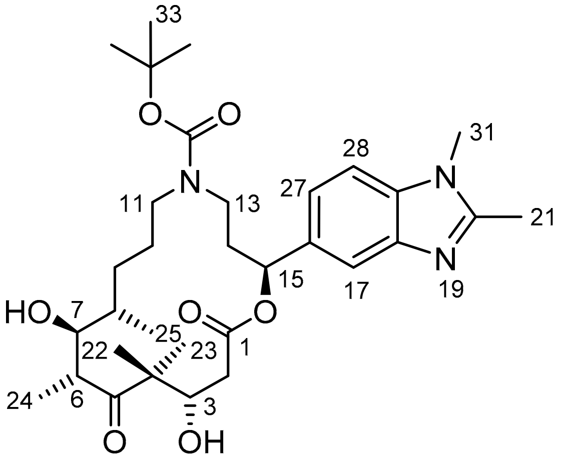

Figure S1. Atom numbering for azathilone **2**.

Table S1. Dihedral angles (°) for **2** free in aqueous solution.

Dihedral Angle	Conformer A	Conformer B
C1C2C3C4	170.2	171.2
C2C3C4C5	−63.3	−61.1
C3C4C5C6	−87.6	−90.8
C4C5C6C7	135.3	134.6
C5C6C7C8	−57.9	−57.0
C6C7C8C9	−66.4	−64.3
C7C8C9C10	174.7	173.2
C8C9C10C11	−153.3	−149.5
C9C10C11C12/C9C10C11N	−171.8	−167.3
C10C11C12C13/C10C11NC13	−76.1	−80.1
C11C12C13C14/C11NC13C14	−73.8	−73.7
C12C13C14C15/NC13C14C15	135.9	129.1
C13C14C15O1	−79.0	−76.3
C14C15O1C1	106.7	116.2
C14C15C16C17	−168.3	9.3

Table S2. NOE-derived interproton distances for azathilone **2** in the tubulin-bound state [1].

Proton Pair	NOE-Based Experimental Distance (Å)	Atomic Distance (Å)	
		Conformer A	Conformer B
2–3	2.8	2.9	2.9
2–7	4.2	4.3	4.3
2–22	4.4	4.3	4.3
2–23	3.1	3.0	3.0
3–6	2.9	2.9	3.0
3–22	2.7	2.8	2.8
3–23	3.6	3.5	3.5
6–7	3.1	3.0	3.0
6–23	4.1	4.1	4.1
6–24	3.0	2.7	2.7
7–24	3.1	3.2	3.2
8–24	4.0	4.2	4.2
13a–15	3.6	3.7	3.7
13b–15	4.0	3.9	3.9
14–15	3.1	2.5	2.5
14–17	3.9	4.3	2.8/4.0
27–14	3.8	3.3	>4.0
15–17	3.0	3.7	2.4
15–27	3.3	2.5	3.7
21–31	3.0	3.0	3.0
22–23	3.5	2.5	2.5
22–24	3.6	3.6	3.6
27–28	2.5	2.5	2.5
28–31	3.2	3.1	3.1

[1] Experimental distances (*r*, Å; ±10%) were estimated according to a full matrix relaxation approach from a build up curve analysis of the ROESY data. Distances were calculated from NOEs with a reference distance of 1.75 Å for the geminal protons.

Table S3. Dihedral angles (°) for **2** bound to α/β tubulin heterodimers [1].

Dihedral Angle	Epothilone A (*syn* Conformer)	Azathilone 2 (Conformer B)
C1C2C3C4	−171.7	170.2
C2C3C4C5	−58.6	−63.3
C3C4C5C6	−74.6	−87.6
C4C5C6C7	147.1	135.3
C5C6C7C8	−61.4	−57.9
C6C7C8C9	−68.2	−66.4
C7C8C9C10	168.1	174.7
C8C9C10C11	177.1	−153.35
C9C10C11C12/C9C10C11N	170.9	−171.8
C10C11C12C13/C10C11NC13	−105.7	−76.13
C11C12C13C14/C11NC13C14	-2.8	−73.83
C12C13C14C15/NC13C14C15	98.0	135.97
C13C14C15O1	−75.4	−79.03
C14C15O1C1	149.7	106.74
C14C15C16C17	−27.0	9.3

[1] Values reported are for the lowest energy conformer found during the refinement of the docking solutions with Macromodel. Dihedral angles for epothilone A are from ref. [1].

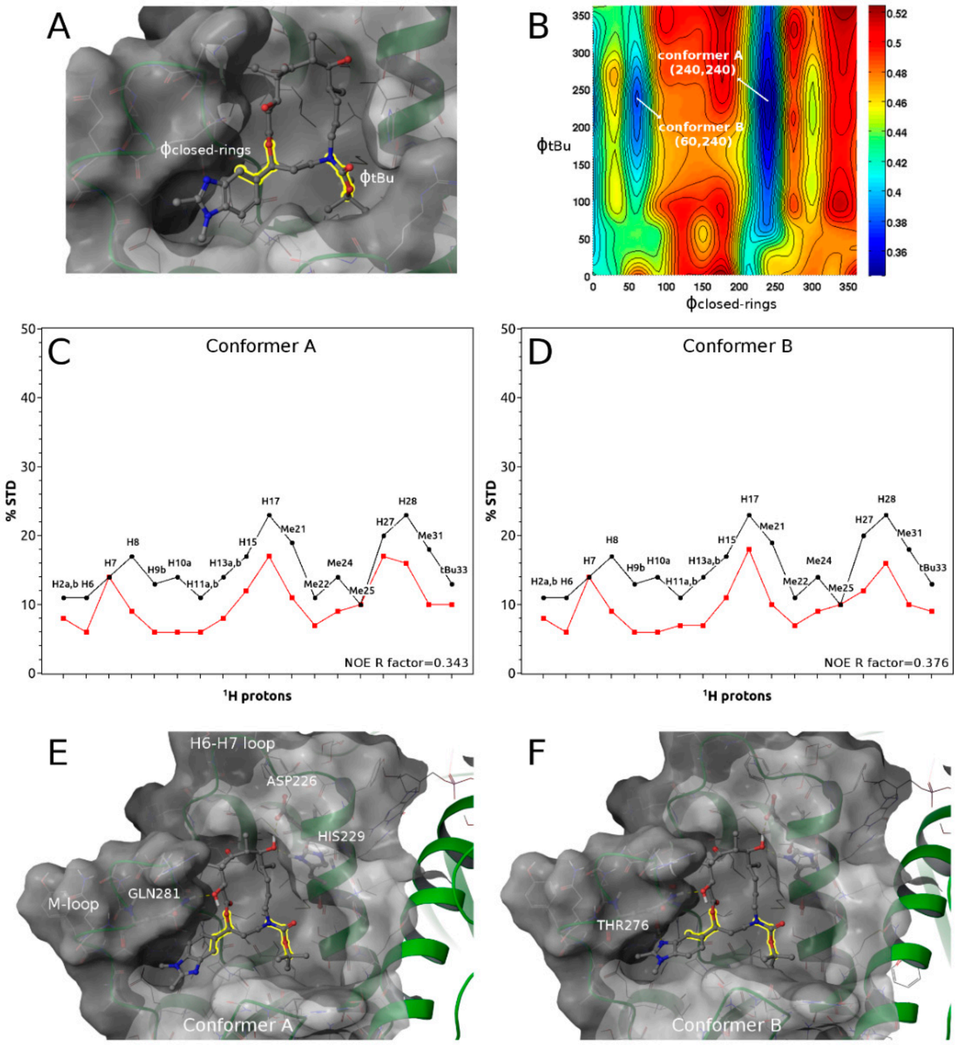

Figure S1. (**A**) Azathilone **2** in the luminal epothilone binding site of β-tubulin; (**B**) NOE R factor landscape obtained from an ensemble of 144 conformers built by torsional scanning of the C15-C16 bond and the *O*-C(CH_3)$_3$ bond in the *t*butyoxycarbonyl substituent; (**C**) Experimental vs. calculated STD profiles for conformer A (black and red, respectively); (**D**) Experimental vs. calculated STD profiles for conformer B (black and red, respectively); (**E**) Refined model of conformer A in complex with β-tubulin; (**F**) Refined model of conformer B in complex with β-tubulin.

Reference

1. Carlomagno, T.; Blommers, M.J.J.; Meiler, J.; Jahnke, W.; Schupp, T.; Petersen, F.; Schinzer, D.; Altmann, K.-H.; Griesinger, C. Structural basis of the activity of the microtubule-stabilizing agent epothilone a studied by NMR spectroscopy in solution. *Angew. Chem. Int. Ed.* **2003**, *42*, 2511–2515.
